# Novel hemagglutinating, hemolytic and cytotoxic activities of the intermediate subunit of *Entamoeba histolytica* lectin

**DOI:** 10.1038/srep13901

**Published:** 2015-09-10

**Authors:** Kentaro Kato, Kazuhide Yahata, Bhim Gopal Dhoubhadel, Yoshito Fujii, Hiroshi Tachibana

**Affiliations:** 1 Department of Parasitology Institute of Tropical Medicine (NEKKEN), Nagasaki University, 1-12-4 Sakamoto, Nagasaki, 852-8523, Japan; 2 Department of Protozoology Institute of Tropical Medicine (NEKKEN), Nagasaki University, 1-12-4 Sakamoto, Nagasaki, 852-8523, Japan; 3 Department of Clinical Medicine, Institute of Tropical Medicine (NEKKEN), Nagasaki University, 1-12-4 Sakamoto, Nagasaki, 852-8523, Japan; 4 Department of Eco-epidemiology, Institute of Tropical Medicine (NEKKEN), Nagasaki University, 1-12-4 Sakamoto, Nagasaki, 852-8523, Japan; 5 Department of Infectious Diseases, Tokai University School of Medicine, 143 Shimokasuya, Isehara, Kanagawa 259-1193, Japan

## Abstract

Galactose and *N*-acetyl-D-galactosamine (Gal/GalNAc) inhibitable lectin of *Entamoeba histolytica*, a common protozoan parasite, has roles in pathogenicity and induction of protective immunity in mouse models of amoebiasis. The lectin consists of heavy (Hgl), light (Lgl), and intermediate (Igl) subunits. Hgl has lectin activity and Lgl does not, but little is known about the activity of Igl. In this study, we assessed various regions of Igl for hemagglutinating activity using recombinant proteins expressed in *Escherichia coli*. We identified a weak hemagglutinating activity of the protein. Furthermore, we found novel hemolytic and cytotoxic activities of the lectin, which resided in the carboxy-terminal region of the protein. Antibodies against Igl inhibited the hemolytic activity of *Entamoeba histolytica* trophozoites. This is the first report showing hemagglutinating, hemolytic and cytotoxic activities of an amoebic molecule, Igl.

Amoebiasis caused by infection with *Entamoeba histolytica* (*E. histolytica*) is a particularly problematic parasitic disease in both developing and developed countries. *E. histolytica* causes an estimated 50 million cases of dysentery, colitis and extraintestinal abscesses, resulting in 40,000 to 100,000 deaths annually^1^. Adherence of *E. histolytica* trophozoites to colonic mucins and host cells is essential for colonization, invasion and subsequent pathogenesis, and is mediated by a galactose (Gal)- and *N*-acetyl-D-galactosamine (GalNAc)-inhibitable lectin^2^. The 260-kDa Gal/GalNAc inhibitable lectin is a heterodimer of transmembrane heavy subunit (Hgl, 170 kDa) and glycosylphosphatidylinositol (GPI)-anchored light subunit (Lgl, 35/31 kDa) glycoproteins linked by disulfide bonds. Hgl contains a carbohydrate recognition domain (CRD) and is the key molecule in amebic adherence.

A GPI-anchored 150-kDa intermediate subunit (Igl, 150 kDa) is non-covalently associated with Hgl and also contributes to adherence^3^. A mouse monoclonal antibody (mAb) to Igl significantly inhibits adherence of *E. histolytica* trophozoites to erythrocytes and Chinese hamster ovary (CHO) cells, cytotoxicity of trophozoites to CHO cells, and erythrophagocytosis of trophozoites^4^,^5^. Immunization of hamsters with native Igl and passive immunization with the mAb raised against Igl inhibits amebic liver abscess formation in hamsters^6^,^7^. Igl is also detected in the *E. histolytica* fraction that interacts with the brush border of human enterocytes^8^. There are two isoforms of Igl, which are referred to as Igl1 and Igl2, and both are cysteine-rich proteins containing multiple CXXC motifs^9^. Two Igls also exist in *E. dispar*, which is morphologically indistinguishable from *E. histolytica*, but is non-pathogenic^10^. The expression level of the *Igl1* gene is higher in *E. histolytica* than in *E. dispar*, whereas that of the *Igl2* gene is comparable in the two species, suggesting that Igl1 may be more closely associated with the pathogenicity of *E. histolytica*.

Evaluation of the reactivity of sera from patients with amoebiasis to fragments of Igl1 shows sensitivities of 56%, 92%, and 97% for the N-terminal (N-Igl), middle (M-Igl), and C-terminal (C-Igl) regions, respectively^11^. All sera from asymptomatic patients react with M-Igl and C-Igl, which suggests that antibodies to epitopes located in M-Igl and C-Igl may function to prevent invasion of trophozoites into host tissue. Indeed, a human mAb recognizing N-Igl does not inhibit amebic adherence to CHO cells, whereas another mAb reactive with M-Igl and C-Igl does inhibit adherence^12^. Therefore, it is important to identify the location of the domain related to amebic adherence in Igl.

Igl was identified as a lectin because it binds to a Gal-affinity column composed of *p*-aminophenyl-β-D-galactopyranoside-Sepharose gel^3^. *E. histolytica* Igl has also been detected, in addition to Hgl and Lgl, in the protein fraction that binds to GalNAc-bovine serum albumin-coated magnetic beads^13^. However, the amino acid sequences of both Igls lack a known CRD of other lectins. Since the details of the function of Igl in amebic adherence are unclear, the molecular properties of Igl require investigation. In this study, we evaluated the lectin activity of Igl using a glycan array and also examined the *in vitro* effects of Igl in erythrocytes and Caco-2 cells. These studies revealed novel roles of Igl in the pathogenicity of *E. histolytica*.

## Results

### Recombinant Igls

**Figure 1:**
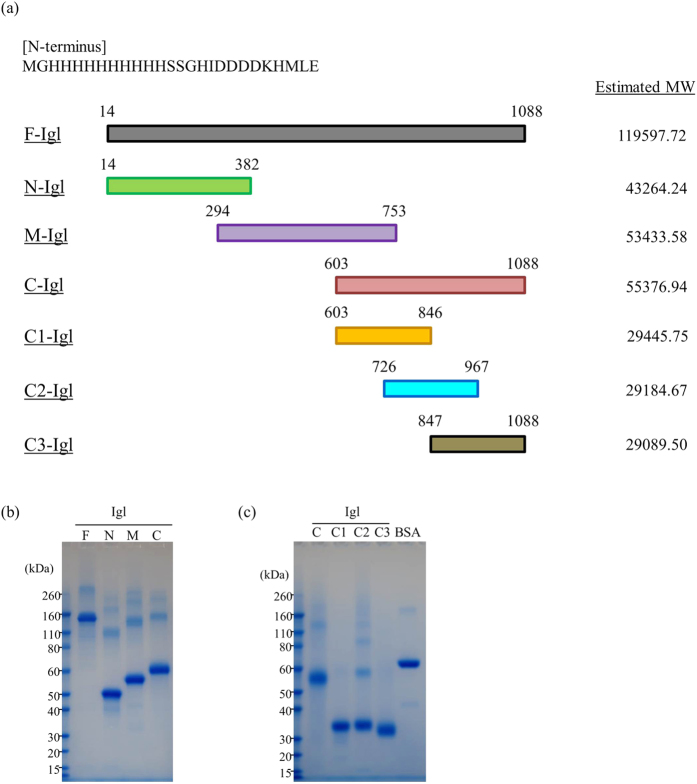
Recombinant Igl proteins used in this study. (**a**) Recombinant Igl proteins were constructed with a His-tag at the N-terminus. Full length (F-Igl), N-terminus (N-Igl), middle (M-Igl) and C-terminus (C-Igl) Igl1 were expressed in *E. coli* and purified using Ni columns. Three C-terminal fragments (C1-Igl, C2-Igl, C3-Igl) were also used in the study. Estimated molecular weights of each protein including the His-tag are shown. (**b**,**c**) The protein purity and amount were confirmed with SDS-PAGE using NuPAGE Novex Bis-Tris (4–12% gradient) gels with 1 μg of each protein.

Full-length Igl (F-Igl, aa 14 to 1088), N-terminal (N-Igl, aa 14 to 382), middle (M-Igl, aa 294 to 753), and C-terminal (C-Igl, aa 603 to 1088) regions, and fragments of C-Igl of *E. histolytica* Igl1 with a His-tag at the N-terminus were expressed in *E. coli*, using the primer sets shown in Table 1^11^. The recombinant proteins were purified using Ni columns, the buffer was changed to PBST, and the sample purities were confirmed by SDS-PAGE (Fig. 1b,c). These recombinant proteins were then used in further studies.

### Hemagglutinating and hemolytic activities of recombinant proteins against horse red blood cells (HoRBCs)

**Figure 2:**
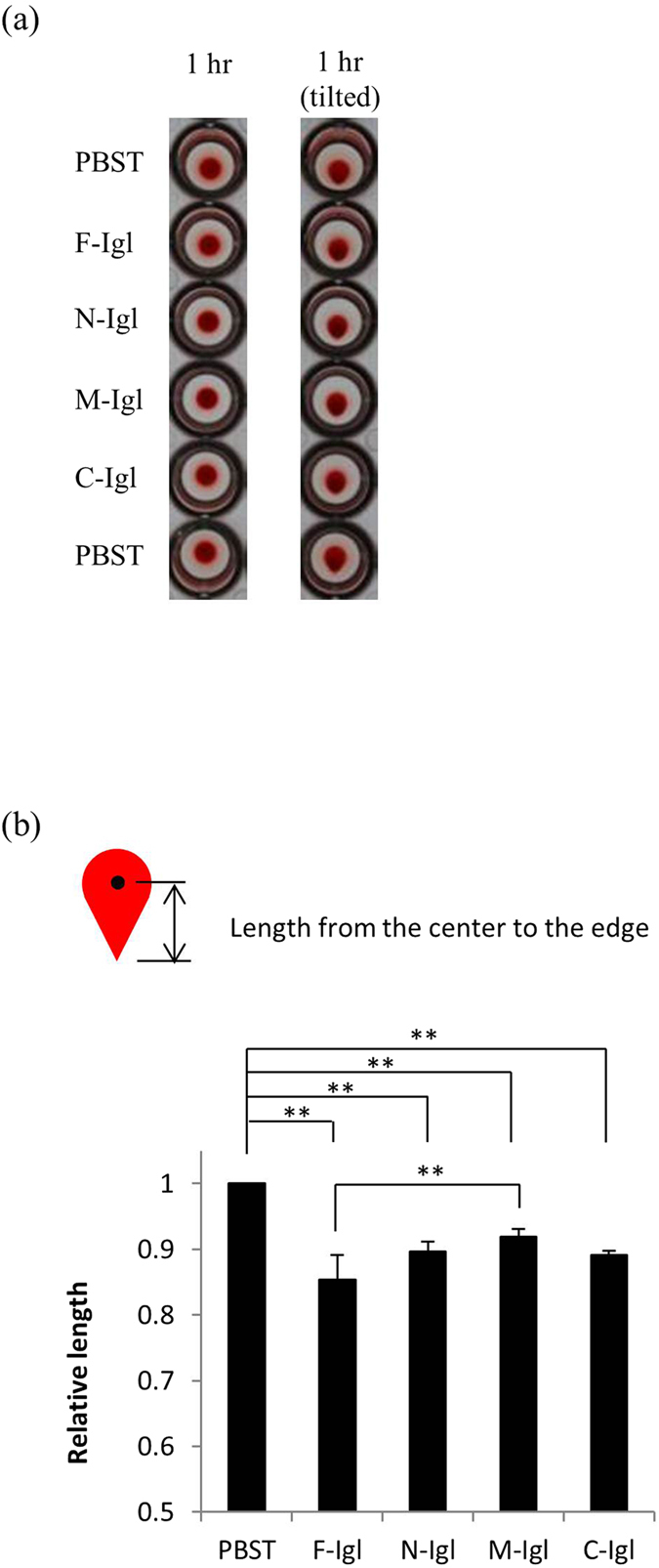
Hemagglutinating activities of Igl proteins in horse red blood cells (HoRBCs). Fifty microliters of 2 μM recombinant Igl proteins were incubated with 50 μl of 2% (v/v) HoRBCs in PBS for 1 h. (**a**) Images of HoRBCs left to stand and tilted in a 96-well plate after 1 h of incubation with recombinant Igls. Representative images are shown from five experiments each. (**b**) Bar graph showing lengths of the HoRBCs from the center to the edge after the plate was tilted. Data are the mean + SD from five independent experiments. **p < 0.01 for multiple comparisons (Dunn’s test).

Since Igl is a lectin, we first wanted to clarify the region of Igl that is responsible for this activity. A commercial glycan array was used to check the lectin activity of F-Igl. As shown in Supplementary Fig. S1 online and Table 2, there was no significant affinity of F-Igl for the glycans on the array. Therefore, an established hemagglutinating assay was used to identify the region responsible for the hemagglutinating activity of Igl. We first determined the optimum concentration of Igls for using in this study. As shown in Supplementary Fig. S2 online, a peanut lectin, PNA, showed its hemagglutinating activity at the concentration of 100 and 200 μg/ml after 1 h of incubation with HoRBCs (2% v/v). At the same concentrations, F-Igl and C-Igl showed hemolytic activities after 18 h of incubations. Protein concentrations of 100 and 200 μg/ml correspond to 0.836 and 1.67 μM of F-Igl protein respectively. Therefore, we used 1 μM proteins for further experiments in this study. HoRBCs (2% v/v) in PBS were mixed with F-Igl, N-Igl, M-Igl or C-Igl (Fig. 2). After the samples were mixed in a 96-well plate with a U-bottom, the plate was incubated at room temperature for one hour to evaluate the hemagglutinating activities of the proteins (Fig. 2a). All recombinant Igl proteins showed some hemagglutinating activities, and to assess these activities the 96-well plate was tilted for 15 s and the lengths of deposited HoRBCs from the center to the edge were measured (Fig. 2b).

**Figure 3:**
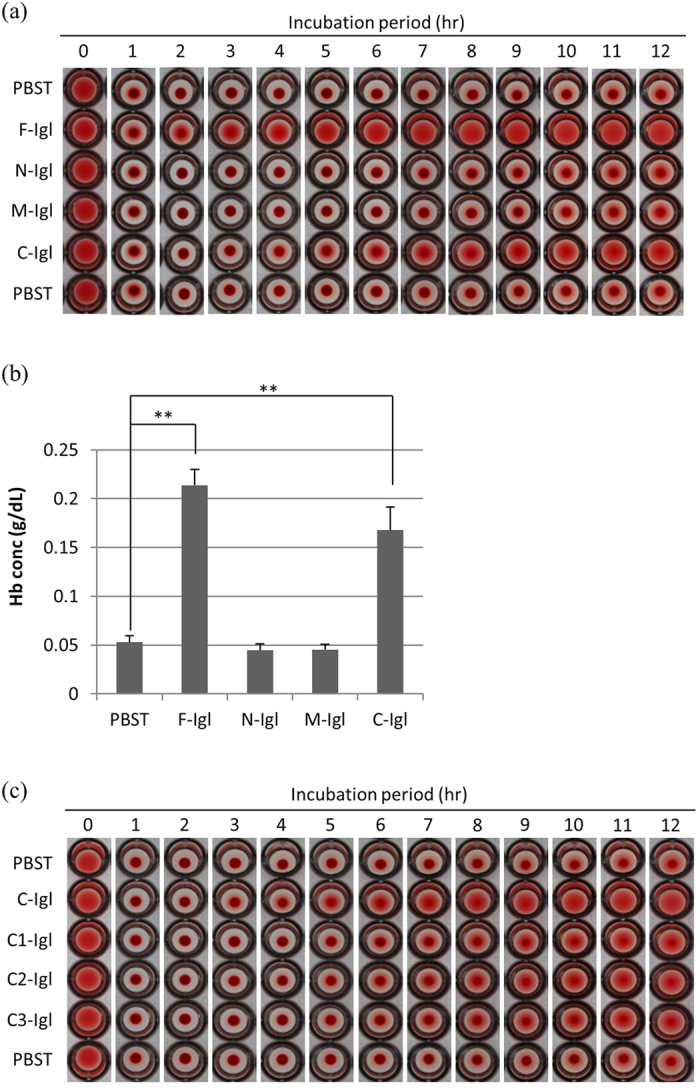
Time-course of hemolytic activities of Igl proteins. Fifty microliters of recombinant Igl proteins (2 μM) were incubated with 50 μl of 2% (v/v) HoRBCs in PBS for the indicated periods. (**a**) Images of HoRBCs in a 96-well plate after the indicated incubation periods with Igls. (**b**) Concentrations of hemoglobin (Hb) released in the supernatant of samples incubated for 8 h. Data are the mean + SD from five independent experiments. **p < 0.01 for PBST vs. Igl treatment (Dunnett’s test). (**c**) Images of HoRBCs in a 96-well plate after the indicated incubation periods with C-Igl, C1-Igl, C2-Igl and C3-Igl.

Incubation of samples up to 12 h was performed to examine differences over a longer incubation period. Both F-Igl and C-Igl showed hemolytic activities after 12 h of incubation, but N-Igl and M-Igl did not do so (Fig. 3). In the time-course assay, the hemolytic activity of F-Igl started to be observed after 2 h of incubation and that of C-Igl after 5 h, while N-Igl and M-Igl showed no activity based on visual observation (Fig. 3a). To show that these results were really due to hemolysis of HoRBCs, the released hemoglobin (Hb) concentration was measured in the supernatant of incubated samples (Fig. 3b). As expected, Hb concentrations in F-Igl or C-Igl treated samples were significantly higher than that after PBST treatment, while those after N-Igl or M-Igl treatment were equivalent to PBST treatment (Fig. 3b).

We tried to narrow down the regions responsible for hemolytic activity by conducting the same hemolysis time-course assay using three fragments of C-Igl (C1-Igl, C2-Igl and C3-Igl) (Fig. 1). All the fragments showed similar hemolytic activities (Fig. 3c), which suggests that C2-Igl (aa 726–967) contains multiple active hemolytic sequences. These results showing that the C-terminal of Igl possesses hemolytic activity were supported by a comparison with the activity of NM-Igl (aa 14–753) (Supplementary Fig. S3 online), a recombinant protein containing the N-terminus and middle of Igl with an 80 kDa molecular weight (Supplementary Fig. S3a and S3b online). In incubation of 1 μM NM-Igl or C-Igl with HoRBCs, both proteins showed hemolytic activity, but C-Igl had twice the activity of NM-Igl after 8 h. This suggests that the C-terminus of Igl has the main hemolytic activity (Supplementary Fig. S3c and S3d online).

### Hemolytic activities of the recombinant proteins against B^+^ human red blood cells (HuRBCs)

**Figure 4:**
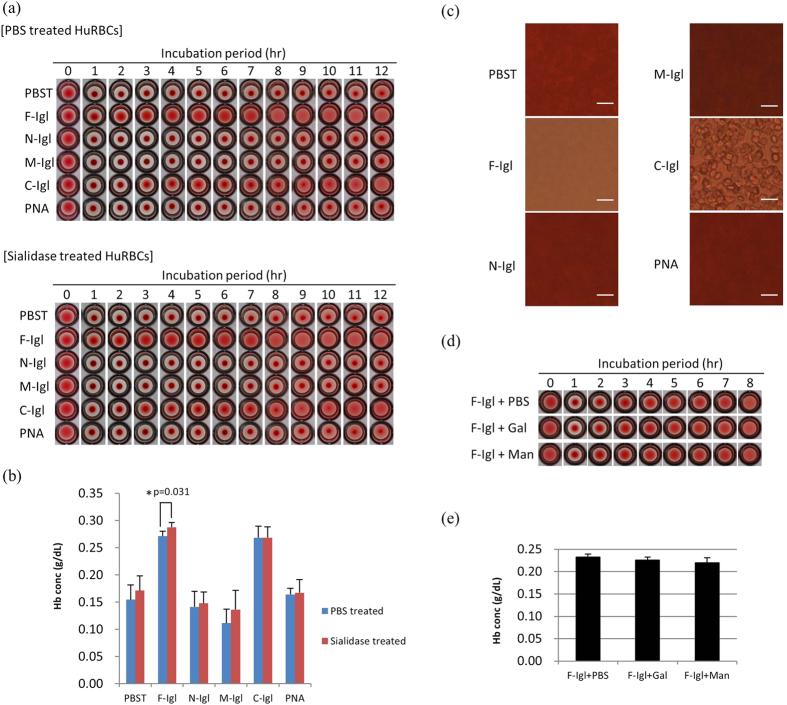
Hemolytic activities of Igl proteins in B^+^ human red blood cells (HuRBCs). Fifty microliters of recombinant Igl proteins (2 μM) were incubated with 50 μl of 2% (v/v) HuRBCs in PBS for the indicated periods. (**a**) Images of HuRBCs in a 96-well plate after the indicated incubation periods with Igls or PNA. HuRBCs used in this study were treated with PBS or sialidase. (**b**) Concentrations of hemoglobin (Hb) released in the supernatant after incubation for 8 h. Data are the mean + SD from five independent experiments. *p < 0.05 for PBS treatment vs. sialidase treatment (Student’s t-test). (**c**) Microscopic observations of HuRBCs after incubation for 8 h with Igls or PNA. Bars indicate 20 μm. (**d**,**e**) Galactose or mannose was mixed with F-Igl prior to mixing with HuRBCs. No significant differences among samples were observed visually during the incubation period or in the concentrations of released Hb after 8 h of incubation (Dunn’s test).

Glycans on the surface of the HoRBCs may have differed among sample lots because we could not assign the blood type of the HoRBCs. To show that the activities of the proteins were not specific for HoRBCs, we conducted the same study using B^+^ human red blood cells (HuRBCs), in which some glycans on the surface had galactose residues at the terminus of the glycans (Fig. 4). F-Igl and C-Igl showed hemolytic activities with HuRBCs (Fig. 4a) that were similar to the results for HoRBCs (Fig. 3a).

Sialic acids on RBCs may also affect the hemolytic activity of the Igl proteins. To examine this possibility, we treated HuRBCs with sialidase prior to the assay. Sialidase treatment of HuRBCs did not affect the hemolytic activities of F-Igl and C-Igl over 12 h of incubation (Fig. 4a). F-Igl and C-Igl induced hemolysis in this assay and that the results were not affected by the presence of sialic acids on HuRBCs. Interestingly, a galactose-recognizing lectin, PNA, showed no hemolytic activity at the same concentration as F-Igl or C-Igl (Fig. 4b). This was confirmed by microscopic observations showing that no or few HuRBCs remained in samples after 8 h of incubation with F-Igl or C-Igl, whereas more HuRBCs remained in other samples (Fig. 4c).

We also assessed whether the hemolytic activity could be inhibited by monosaccharides. Mixing of tenfold higher concentrations of galactose or mannose with F-Igl prior to mixing with HuRBCs produced no significant difference in visual observation and released Hb concentrations (Fig. 4d,e). Collectively, these results indicate that the hemolytic activities of the Igl proteins were not specific to HoRBCs and were unaffected by sialic acid, galactose, and mannose residues on the surface of HuRBCs.

### Cytotoxic activities of recombinant Igl proteins against human colon carcinoma Caco-2 cells

**Figure 5:**
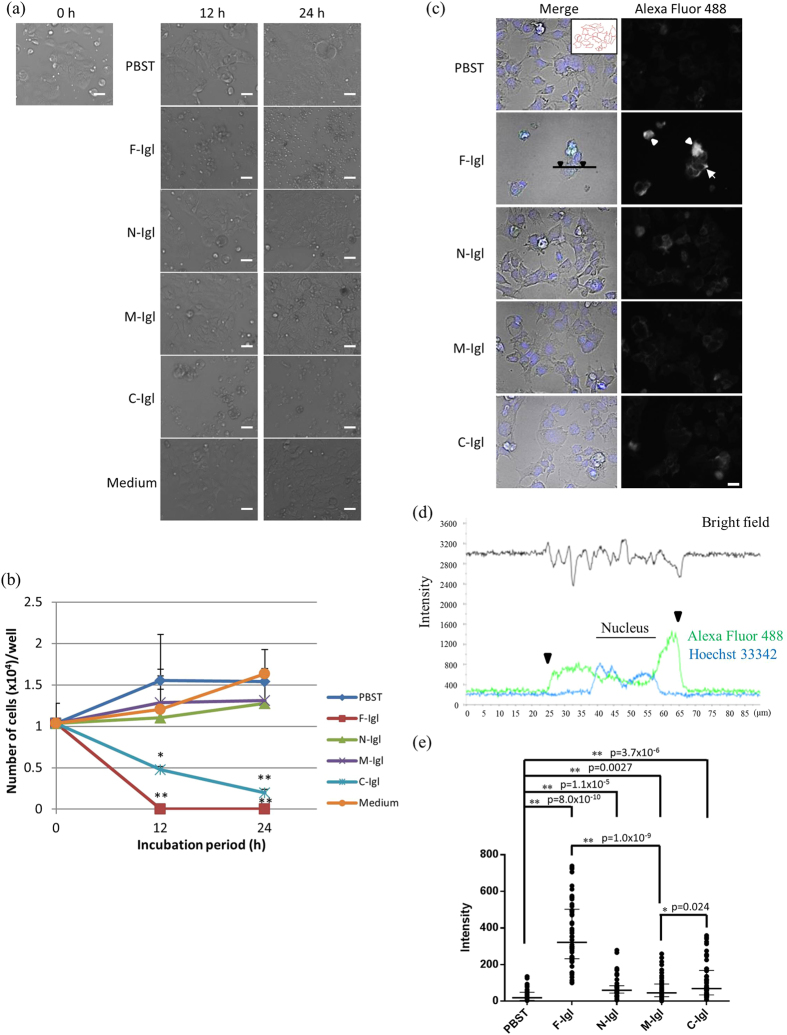
Cytotoxic activities of Igl proteins against human colon carcinoma Caco-2 cells. (**a**,**b**) Caco-2 cells were cultured in a 96-well plate for 24 h prior to incubation with recombinant Igl proteins (2 × 10^4^/100 μl/well). One-hundred microliters of Igl proteins (2 μM) or medium were added and incubated for an additional 12 or 24 h. (**a**) Images of Caco-2 cells in a 96-well plate after the indicated incubation periods with Igls taken with an EVOS-XL microscope. Bars indicate 50 μm. (**b**) Line graph of the number of Caco-2 cells remaining in the well after 0, 12 and 24 h of incubation. Data are the mean + SD from five independent experiments. *p < 0.05, **p < 0.01 for medium vs. Igl treatment (Dunnett’s test). (**c**–**e**) Caco-2 cells were spread into an ibidi 6-well flow chamber and incubated with Igl proteins. (**c**) Alexa Fluor 488 fluorescence of Caco-2 cells treated with PBST, F-Igl, N-Igl, M-Igl, or C-Igl. Cells were isolated in a bright field image with Hoechst 33342 staining (inset in PBST treated cells) for measuring the median fluorescence intensity of each cell in further studies. Open arrowheads and an open arrow indicate round cells and the edge of an attached cell, respectively, for which strong F-Igl attachment was observed. The open bar indicates 20 μm. (**d**) Fluorescence signal intensities of a F-Igl treated cell measured as shown in panel C with closed arrowheads indicating the edges of the cell. (**e**) Quantitative comparison of fluorescence intensities detected by fluorescence microscopy. Fifty cells were randomly selected for each group. The median fluorescence of each group is shown with interquartile ranges. *p < 0.05, **p < 0.01 between the groups (Wilcoxon signed-rank test).

The results above show that Igl has novel hemolytic activity with HoRBCs or HuRBCs. To evaluate whether Igl is also cytotoxic, human colonic epithelial Caco-2 cells were cultured with recombinant Igl proteins. Caco-2 cells proliferated with a cobblestone appearance after 12 or 24 h of incubation with PBST, N-Igl, M-Igl or medium, but became round and died when cultured with F-Igl or C-Igl (Fig. 5a). There was a significant decrease in the number of cells remained on the plate in incubation with F-Igl or C-Igl (Fig. 5b). The viability of the cells was assessed by the trypan blue exclusion assay. Attached and detached cells were confirmed as dead cells in the F-Igl treated group after 12 h of incubation (Supplementary Fig. S4 online).

*E. histolytica* has contact-dependent cytotoxicity against host cells and contact-dependent transfer of *E. histolytica* lectins to host cells can occur^14^. Therefore, we examined if the recombinant Igl proteins could attach to Caco-2 host cells by incubating proteins labeled with Alexa Fluor 488 with Caco-2 cells (Fig. 5c). Interestingly, F-Igl attached strongly to the entire round shaped cell and the signal was also observed at the edge of the adherent cells (Fig. 5d). Igl-treated cells showed a significant increase in fluorescence signal intensities compared to PBST-treated cells (Fig. 5e: PBST: median 19.0 (IQR 44.4), F-Igl: 321.7 (270.6), N-Igl: 59.6 (40.4), M-Igl: 45.7 (69.2), C-Igl: 68.9 (134.2)). The higher median values for F-Igl- and C-Igl suggest that these proteins attached to Caco-2 cells (Fig. 5e) and thereby caused cell death. The lower attachment of C-Igl compared to that of F-Igl may be due to the short (1 h) incubation. A delay in the activity of C-Igl compared to F-Igl is consistent with the results in the hemolytic (Figs 3 and 4) and cytotoxicity (Fig. 5) assays. Collectively, our results show that Igl has novel hemagglutinating, hemolytic and cytotoxic activities *in vitro*.

### Inhibition of hemolytic activity of Entamoeba histolytica trophozoites by anti-Igl monoclonal antibodies

**Figure 6:**
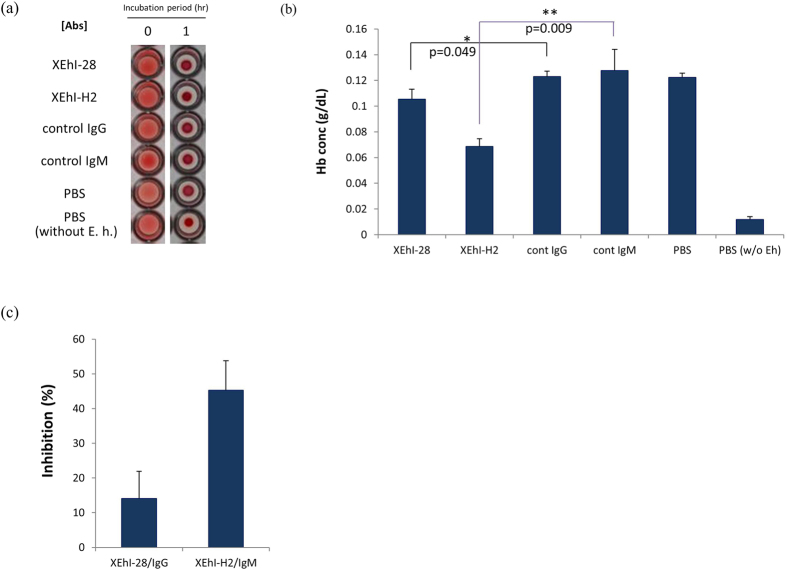
Inhibition of hemolytic activity of *Entamoeba histolytica* trophozoites by anti-Igl monoclonal antibody treatments. Ten micrograms of anti-Igl antibodies (XEhI-28 and XEhI-H2) or control antibodies were incubated with 5 × 10^5^ *E. histolytica* trophozoites prior to hemolytic assay. (**a**) Images of HoRBCs in a 96-well plate after the indicated incubation periods with trophozoites or PBS. (**b**) Concentrations of hemoglobin (Hb) released in the supernatant after incubation for 1 h. Data are the mean + SD from three independent experiments. *p < 0.05, **p < 0.01 for anti-Igl mAb vs. isotype control Ab treatment (Student’s t-test). (**c**) Percentage inhibition of hemolytic activity of the trophozoites by anti-Igl mAb treatments. The values are 14 ± 7.9% for XEhI-28/IgG and 45.3 ± 8.5% for XEhI-H2/IgM.

To assess whether these activities reflect the activities of intact Igl on *E. histolytica* trophozoites, monoclonal antibodies recognizing N-Igl (XEhI-28) and M/C-Igl (XEhI-H2) were incubated with the trophozoites prior to the hemolytic assay (Fig. 6). Significant inhibition in hemolytic activity was observed when the trophozoites were pre-incubated with anti-Igl antibodies (Fig. 6b). About 45% (45.3 ± 8.5) inhibition in the activity was observed when the XEhI-H2 antibody-treated group was compared with the isotype control-treated group. XEhI-28 antibody-treated group showed around 15% (14.1 ± 7.9) inhibition compared with control IgG-treated group indicating that middle or C-terminus region of intact Igl on *E. histolytica* trophozoites has the main hemolytic activity (Fig. 6c).

## Discussion

The *E. histolytica* lectin consists of three subunits, Hgl, Lgl and Igl, of which Hgl and Igl have lectin activities^15^. Little is known about the lectin activity of Igl, except that this protein binds to a Gal-affinity column^3^. In this study, we planned to determine the regions of Igl that have lectin activity, but glycan array results did not indicate any lectin activity of Igl and the hemagglutinating activity of the protein was weak. However, novel hemolytic activity was found that was associated with the C-terminus of Igl, and the protein was also cytotoxic in Caco-2 cells. Furthermore, hemolytic activity of *E. histolytica* trophozoites was inhibited by monoclonal antibodies against Igl. To our knowledge, this is the first study to show that *E. histolytica* Igl lectin has hemagglutinating, hemolytic and cytotoxic activities.

Involvement of Gal/GalNAc lectin of *E. histolytica* in cytotoxicity towards host cells has been studied for several decades. This is because the activity can be inhibited by GalNAc^16^ or monoclonal antibody raised against Hgl^17^. The *E. histolytica* membrane also has hemagglutinating activity toward human A^+^ red blood cells^18^. However, despite Hgl having a CRD^19^, involvement of Hgl in this activity has not been examined. The unexpected hemagglutinating, hemolytic, and cytotoxic activities of Igl suggests the presence of a CRD, but we were not able to identify a CRD in Igl using a glycan array (Table 2 and Supplementary Fig. S1 online), even though Igl binds to a Gal-affinity column^3^. This might be because the affinity of Igl for glycans on the array is weak. Hgl has low affinity for a single GalNAc residue compared to that for multiple GalNAc-attached neoglycoproteins^20^,^21^, and this may also be the case for Igl. In fact, in our preliminary study, we could observe affinities of F-Igl and C-Igl toward (GalNAc)_19_-HSA (Supplementary Fig. S5a online). However, all recombinant Igl proteins had also high affinities toward HSA (Supplementary Fig. S5b online). More intensive studies need to be done to conclude the region of Igl having the lectin activity.

Two isoforms of Igl are also present in non-pathogenic *E. dispar*, with amino acid sequence identities of 75–76% for Igl1 and 73–74% for Igl2 between *E. histolytica* and *E. dispar*. However, the expression level of Igl1 is higher in *E. histolytica* than in *E. dispar*^10^. This suggests that Igl1 may have a role in the pathogenicity of *Entamoeba*, but it is important to emphasize that Igl was originally identified using monoclonal antibodies specific for *E. histolytica*^22^. Regardless, it will be important to examine whether the expression level of Igl correlates with the virulence of *E. histolytica* in more detail.

The name “*histolytica*” was given to this parasite because it destroys host tissues in a contact-dependent manner. One of the best-known amoebic proteins responsible for this effect is amoebapore, a pore-forming protein that also possesses cytolytic activity but not hemolytic activity towards human or rabbit erythrocytes^23^,^24^. Amoebapore is present in amoeba granules and requires stimulatory signals for its secretion for contact-mediated cytolysis of host cells. The primary function of the protein is thought to be the killing of ingested bacteria or cells inside their digestive vacuoles^24^,^25^. Furthermore, a higher concentration of amoebapores (>10–100 μM) is required for their cytotoxic activities^26^. In contrast, 1 μM Igl was required for activity in the present study. Igl1 and Igl2 are co-localized on the surface and cytoplasm of the parasite, but different localization patterns in intracellular vacuoles have also been detected^10^,^12^. *E. histolytica* phagosomes contain Igl and the quantity of Igl varies during maturation of the phagosome^27^,^28^. Therefore, the hemolytic and cytolytic activities of Igl may also be important in the killing of ingested cells in the phagosome. In another study, an *Entamoeba histolytica* strain lacking amoebapore-A (the G3 strain) showed reduced target cell destruction, cytotoxic and hemolytic activities compared with a parental HM-1:IMSS strain^29^, which indicates that expression of amoebapore-A is required for the hemolytic activity of the HM-1:IMSS strain. It is of note that, in the same study, an avirulent Rahman strain showed a similar phenotype to that of the G3 strain, even though the Rahman strain expressed similar protein levels of amoebapore-A compared with the HM-1:IMSS strain. This indicates involvement of other factors in these phenotypes.

The amino acid sequence of amoebapore-A^30^ was aligned with those of the C1-, C2-, and C3-Igls to obtain information on the hemolytic activity of Igl (Supplementary Fig. S6 online). There were some similar sequences in amoebapore-A and the C-Igl fragments, but no conserved motif that might contribute to Igl activity. A unique pore-forming protein from *Acantamoeba culbertsoni* has recently been described^31^ as an acanthaporin of the saposin-like protein (SAPLIP) family, similarly to amoebapore-A. As shown in Supplementary Fig. S7 online, this protein also had no conserved domain with the C-Igl fragments, especially with the C2-Igl sequence, in which the hemolytic activity of Igl seemed to reside (Fig. 3c). A marine invertebrate, *Cucumaria echinata*, has a Ca^2+^-dependent Gal/GalNAc lectins (CEL-I-IV)^32^, among which CEL-III exhibits strong hemolytic and cytotoxic activities^33^,^34^. Since *E. histolytica* Igl also possesses hemolytic and cytotoxic activities, we compared the CEL-III amino acid sequence^35^ with those of the C-Igl fragments (Supplementary Fig. S8 online). Clusters of homologous amino acid sequences were found (Supplementary Fig. S8a-c online) with conserved CX_8_C motifs among these sequences (Supplementary Fig. S8, boxes ①–⑦ online). An amino acid important for the hemolytic activity of CEL-III, Arg408, is in one of these CX_8_C motifs. Comparison of Igl with Hgl showed that Hgl does not have CX_8_C motifs in the comparable region (Supplementary Fig. S8d online). F-Igl contains 9 CX_8_C motifs and N-Igl, M-Igl, and C-Igl have 1, 1, and 7 motifs, respectively. The C2-Igl and C3-Igl fragments contain several motifs (Supplementary Fig. S8a-c online), but C1-Igl has only one. More importantly and interestingly, these motifs exist in *E. dispar* Igl1 and Igl2^10^. As mentioned above, the expression level of Igls might correlate with the virulence of *E. histolytica*. Thus, a further study is needed to determine whether these motifs are important for the activities of Igl.

There is also a need to determine if Igl possesses the hemolytic and cytotoxic activities of the parasite and has a role at the site of infection *in vivo*. These findings will emerge from future studies using attenuated *E. histolytica* that lacks Igl protein.

## Methods

### Expression and refolding of recombinant F-Igl, N-Igl, M-Igl, C-Igl and fragment C-Igl proteins

*E. coli* BL21 Star(DE3)pLysS cells (Invitrogen) or ECOS^TM^ competent BL21(DE3) cells (Nippon Gene Co.) were transformed with plasmids containing a DNA fragment coding F-Igl, N-Igl, M-Igl, C-Igl or C-Igl fragments (C1-, C2- or C3-Igl) ligated with a pET19b vector (Novagen), as described previously^11^. The primer sets are shown in Table 1. The gene-transformed BL21 Star(DE3)pLysS cells were cultured in 2 ml of LB medium containing ampicillin (100 μg/ml) and chloramphenicol (34 μg/ml) in a multi-shaker incubator at 37 °C overnight. BL21(DE3) cells were cultured under the same conditions, except for the addition of chloramphenicol. The turbid culture was transferred to 2 × YT medium and incubated at 37 °C until the optical density (OD_600_) reached 0.6. Isopropyl-β-D-thiogalactopyranoside (0.25 mM) was added to the culture, incubated at 22 °C for 20 h, and centrifuged at 6500 × g for 15 min. The pellet of *E. coli* was washed with 1 × IB Wash Buffer (20 mM Tris-HCl pH 7.5, 10 mM EDTA, 1% Triton X-100) from a Protein Refolding Kit (Novagen) and resuspended in the same buffer supplemented with Benzonase Nuclease (Sigma, 1 μl/100 ml culture). For BL21(DE3) cells, 100 μg/ml of Lysozyme (Thermo) were added to the samples and incubated at 30 °C for 15 min. Sonication of the samples was conducted using a Sonifier SLPe Digital Ultrasonic Homogenizer (Branson) or a USP-400 A ultrasonic breaker (Shimadzu Co., Ltd.). The samples were centrifuged at 10,000 × g for 10 min at room temperature. C2-Igl recombinant protein was obtained from the supernatant of homogenized samples and applied to a Ni column without solubilizing and dialyzing steps. Solubilization and refolding of other recombinant proteins were conducted according to the instructions provided with the Protein Refolding Kit (Novagen).

### Ni column purification of recombinant proteins

The His-tagged refolded proteins were further purified using a Ni column. Five milliliters of the protein solution was batched with 1 ml of Ni-NTA agarose (Qiagen) at 4 °C overnight. The mixtures were loaded onto hand-made columns. The column was washed three times with 1 ml of PBS containing 10 mM imidazole. Recombinant proteins were eluted with PBS containing 200 mM imidazole (1 ml/fraction). The fractions containing recombinant proteins were pooled and buffers were changed to PBST (PBS with 0.05% Tween 20) with Amicon Ultra Ultracel-10 K (Millipore) before further use. The protein concentration was determined using BioRad protein assay reagent.

### SDS-PAGE and Coomassie Brilliant Blue staining of purified recombinant proteins

Recombinant proteins (1 μg each) were mixed with a one-third volume of SDS sample buffer (Invitrogen) and subjected to SDS-PAGE using a NuPAGE Novex Bis-Tris (4–12% gradient) gel in reducing condition. Electrophoresis was conducted for 40 min at 200 V. The gel was treated with SimplyBlue Safe stain solution (Invitrogen) and incubated until blue bands appeared on the gel.

### Assay of lectin activity using a glycan array

The lectin activity of F-Igl was measured using a glycan array (Glycan Array I, BS-X1731, Sumitomo Bakelite Co., Japan). Seventy microliters of 200 μg/ml F-Igl was loaded on the array and incubated with mouse anti-His tag antibody (70796–3, Novagen) and Cy5 goat anti-mouse IgG (H + L) antibody (A105247, Life Technologies), following a protocol supplied by Sumitomo Bakelite Co. Affinity signals were measured by the fluorescence of Cy5 and data are expressed as a signal/noise (S/N) value. S/N values >3 were considered to indicate significant binding of F-Igl to glycans. The glycan structures coated on the array are shown in Supplementary Fig. 1b online. This array included areas of high (0.5 mg/ml GAGs, 0.2 mM *N*-glycans, and 1.0 mM other glycans) and low (0.1 mg/ml GAGs, 0.04 mM *N*-glycans, and 0.2 mM other glycans) concentrations of glycans.

### Hemagglutination and hemolytic assays using recombinant lectins

Fifty microliters of recombinant Igls or PNA lectin (2 μM each) were loaded onto a 96U microwell plate (Nunc). Horse or B^+^ human red blood cells (HoRBCs or HuRBCs) were prepared from horse blood in Alsever’s buffer (Nippon Biotest Laboratories Inc., Japan) or purchased from Tennessee Blood Services (Memphis, TN). Two percent (v/v) RBCs in PBS were prepared and 50 μl/well of the RBC solutions were added to the lectin-preloaded wells at room temperature. To assess weak hemagglutinating activities, the 96 multiwell plate was tilted for 15 s and the lengths of deposited HoRBCs from the center to the edge were measured. The results are shown as the mean of 5 experiments with a standard deviation (SD). To evaluate the effect of sialic acids on RBCs on hemolytic activity of the lectins, 1% (v/v) sialidase from Vibrio cholerae (Roche Applied Science) or PBS was added to 2% (v/v) HuRBCs and incubated at 37 °C overnight. Sialidase- or PBS-treated HuRBCs were washed with PBS twice before the assay. Galactose or mannose (20 μM) was added to 50 μl of F-Igl (2 μM) to assess whether the activities were mediated by the lectin domain of the protein. Fifty microliters of 2% (v/v) HuRBCs was added and the samples were incubated at room temperature for up to 8 h. Hemagglutinating and hemolytic activities of the lectins were observed after RBC loading and images were taken at several time points. Hemolytic activities of the lectins were further assessed by observing RBCs under an EVOS XL microscope (Invitrogen).

### Hemolytic assay using Entamoeba histolytica trophozoites

Trophozoites of *E. histolytica* (HM-1:IMSS strain) were grown in TYI-S-33 medium at 37 °C. Monoclonal antibodies against Igl (XEhI-28 and XEhI-H2) and isotype controls^12^ were prepared and used to evaluate the role of intact Igl in the hemolytic activity. This assay was conducted as previously described^36^ with slight modifications. Briefly, 5 × 10^5^ trophozoites were treated with 10 μg of antibodies or PBS and incubated at 4 °C for 1 h. After washing the trophozoites with PBS, the trophozoites were mixed with 1% HoRBCs in 100 μl PBS and incubated at 37 °C for 1 h. The cell suspension was sedimented at 2000 rpm for 5 min and the concentration of hemoglobin in the supernatant of the sample was determined.

### Detection of released hemoglobin concentration

To quantify the hemolytic activity of each recombinant protein, a Hemoglobin B Test Kit (Wako, Osaka, Japan) was used to measure the concentration of hemoglobin in the supernatants of the RBCs and recombinant protein or trophozoite mixtures described above after incubation for 8 h or 1 h. The results are expressed as the mean of 5 or 3 experiments with SD.

### Cytotoxic assay using recombinant lectins

Caco-2 cells (ATCC, HTB-37) were cultured in MEM basic medium (Gibco) supplemented with Earle’s salts, L-glutamine and 20% fetal bovine serum. After detachment with 0.25% Trypsin-EDTA (Gibco), the cells were cultured in a 96-well plate at approximately 2 × 10^4^ cells/100 μl/well at 37 °C under 5% CO_2_ for 24 h. One hundred microliters of 2 μM recombinant Igl, PBST or medium containing 200 units/ml penicillin G and 200 μg/ml streptomycin (Wako, Japan) was added and the cells were incubated for an additional 12 or 24 h under the same conditions. The cells were trypsinized and harvested after 0, 12 or 24 h of incubation and the number of cells per well was counted. The results are shown as the mean of 5 experiments with SD.

### Attachment of recombinant Igl lectins to Caco-2 cells

Caco-2 cells (1 × 10^5^ cells/100 μl/well) were spread into a μ-Slide VI^0.4^ ibiTreat 6-well flow chamber (ibidi GmbH, Germany) and incubated at 37 °C under 5% CO_2_ for 24 h. Cells were washed 3 times with 100 μl of PBS and treated with 100 μl of PBST, 1 μM of F-Igl, N-Igl, M-Igl or C-Igl, or control EMEM medium supplemented with 100 ng/ml Hoechst 33342 (Life Technologies, CA), 100 units/ml penicillin G and 100 μg/ml streptomycin (Wako, Japan). The samples were incubated at room temperature in the dark for 1 h. After washing with PBS, the cells were fixed with 100 μl/well of 4% paraformaldehyde/glutaraldehyde solution at room temperature in the dark for 15 min. The cells were washed again with PBS three times, followed by incubation with 100 μl of 1:1000 diluted His-tag monoclonal antibody (mouse IgG1, Novagen, 70796–3) at room temperature in the dark for 1 h. After washing with PBS, the cells were stained with a 1:1000 diluted Alexa Fluor 488 goat anti-mouse IgG1 antibody (Life Technologies, A21121) at room temperature in the dark for 1 h. The samples were then mounted with 100 μl of ProLong Gold antifade solution (Life Technologies) after washing with PBS three times. Alexa Fluor 488 and Hoechst 33342 fluorescence of the stained cells were captured under a fluorescence microscope (Ti-E, Nikon, Japan) equipped with a ×40 objective lens (Nikon) and a CCD camera (ORCA-R2, Hamamatsu Photonics, Japan). Fifty cells in each group were randomly selected, and Alexa Fluor 488 fluorescence of these cells was analyzed using NIS-Elements Advanced Research imaging software (Nikon). Median fluorescence intensities were calculated with an interquartile range (IQR), compared among the groups, and plotted using GraphPad Prism6 software (GraphPad Software, Inc., La Jolla, CA).

### Statistical analysis

For multiple comparisons, an ANOVA with Dunn’s test or Dunnett’s test was employed. A Student’s t-test and a Wilcoxon signed-rank test were also used in statistical analyses, with P < 0.05 considered significant.

## Additional Information

**How to cite this article**: Kato, K. *et al.* Novel hemagglutinating, hemolytic and cytotoxic activities of the intermediate subunit of *Entamoeba histolytica* lectin. *Sci. Rep.* **5**, 13901; doi: 10.1038/srep13901 (2015).

## Supplementary Material

Supplementary Information

## Figures and Tables

**Table 1:** Oligonucleotide primers used in this study.

Primer	Positions^a^	Sequence (5′ to 3′)^b^	Ref.
EhIgl-S14	40–59	CCCTCGAGGATTATACTGCTGATAAGCT	*
EhIgl-S294	880–898	CCCTCGAGACAGAAGAAAATAAATGTA	*
EhIgl-S603	1807–1827	CCCTCGAGGAAGGACCAAATGCAGAAGAT	*
EhIgl-S726	2176–2195	CCCTCGAGCCATGTCCTGCAAAATGTAA	**
EhIgl-S847	2539–2558	CCCTCGAGACATGTTCAGATAAAGACAC	**
EhIgl-AS382	1129–1146	CCCTCGAGTTAAAGTTTGCATGGTCCATC	*
EhIgl-AS753	2244–2259	CCCTCGAGTTATAGCCTTTGTTCAGTG	*
EhIgl-AS846	2521–2538	CCCTCGAGTTATGCACATTTATCATCACA	**
EhIgl-AS967	2884–2901	CCCTCGAGTTAACATTTAGTACATTCTTC	**
EhIgl-AS1088	3247–3264	CCCTCGAGTTAAATGCCTTTAGCTCCATT	*

^a^Nucleic acid numbering is based on the *E. histolytica Igl1* gene sequence (AF337950).

^b^Nucleotides added for cloning and translation termination are underlined.

^*^Tachibana *et al.*, J. Clin. Microbiol. 2004;42:1069-1074.

^**^This study.

**Table 2:**
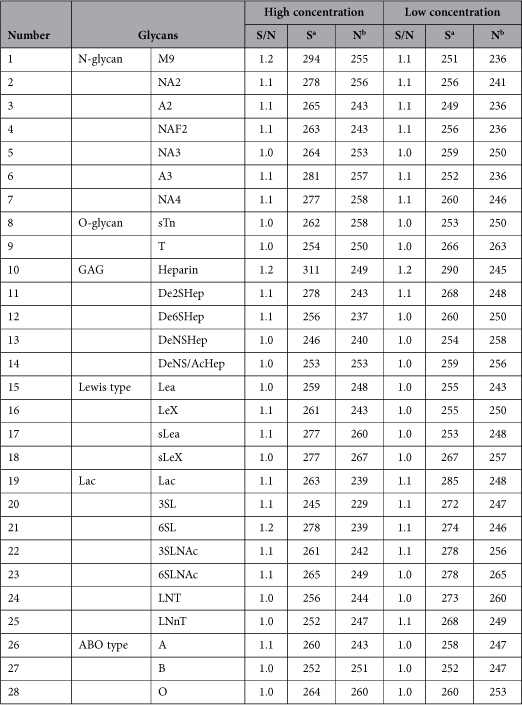
Affinity of recombinant F-Igl for 28 glycans.^c^

^a^Signal. ^b^Noise. ^c^Refer to Supplementary Fig. S1 online for details.
