# Supplementary material for: Novel hemagglutinating, hemolytic and cytotoxic activities of the intermediate subunit of Entamoeba histolytica lectin
Source: Sci Rep. 2015 Sep 10;5:13901. doi: 10.1038/srep13901 (PMC4564812; doi:10.1038/srep13901)
Supplement: Supplementary Information [file srep13901-s1.pdf]

**Novel hemagglutinating, hemolytic and cytotoxic activities of the intermediate subunit of *Entamoeba histolytica* lectin**

Kentaro Kato, Kazuhide Yahata, Bhim Gopal Dhoubhadel, Yoshito Fujii, and Hiroshi Tachibana

## Methods for Supplementary Data

*Assay of lectin activity using a glycan array*

Lectin activity of F-Igl was measured using a glycan array (Glycan Array I, BS-X1731, Sumitomo Bakelite Co., Ltd., Tokyo, Japan). Seventy microliters of 200 μg/ml F-Igl was loaded on the array and incubated with mouse anti-His tag antibody (70796-3, Novagen) and Cy5 goat anti-mouse IgG(H+L) antibody (A105247, Life Technologies) following a protocol supplied by Sumitomo Bakelite Co. Affinity was measured by the fluorescence of Cy5 and data are expressed as a signal-to-noise (S/N) value. S/N values >3 were considered to indicate significant binding of F-Igl to the glycans. The glycan structures coated on the array are shown in Supplementary Fig. 1b online.

*Dose response experiments for hemagglutinating and hemolytic assays*

Two-fold serial diluted lectins (from 200 to 6.25 μg/ml) in PBST (total volume of 50 μl) were loaded on to a 96U Nunclon microwell plate (Nunc). Horse red blood cells (HoRBCs) were prepared from horse blood in Alsever's buffer (Nippon Biotest Laboratories Inc., Tokyo, Japan). HoRBCs in PBS (2% v/v) were prepared and 50 μl/well of the RBC solution was added to the lectin preloaded wells at room temperature. Hemagglutinating activity (1 h) and hemolytic activity (18 h) were observed.

*Expression and refolding of recombinant NM-Igl protein*

ECOS$^{TM}$ competent BL21(DE3) cells (Nippon Gene Co.) were transformed with plasmids containing a DNA fragment coding NM-Igl (aa 14 to 753 of Igl) ligated in a pET19b vector (Novagen). The primer sets are shown in Table 1. The gene transformed BL21(DE3) cells were cultured in 2 ml of LB medium containing ampicillin (100 μg/ml medium) in a multi-shaker incubator at 37°C overnight. The turbid culture of *E. coli* was transferred to a 2×YT medium and incubated at 37°C until the optical density ($OD_{600}$) reached 0.6. Isopropyl-β-D-thiogalactopyranoside (IPTG, 0.25 mM) was added to the culture, incubated at 22°C for 20 h, and centrifuged at 6500×g for 15 min. The pellet of *E. coli* was washed with 1×IB Wash Buffer (20 mM Tris-HCl pH 7.5, 10 mM EDTA, 1% Triton X-100) from a Protein Refolding Kit (Novagen) and resuspended in the same buffer supplemented with Benzonase (Sigma, 1 μl/100 ml culture). To the sample, 100 μg/ml of Lysozyme (Thermo) was added and incubated at 30°C for 15 min. Sonication of the sample was conducted using a Sonifier SLPe Digital Ultrasonic Homogenizer (Branson) and the sample was centrifuged at 10,000×g for 10 min at room temperature after it was no longer viscous. The pellets of inclusion bodies of the recombinant protein were suspended with Solubilization Buffer (50 mM CAPS, pH 11.0, supplemented with 0.3% *N*-lauroylsarcosine and 1 mM DTT), incubated at room temperature for 15 min, and centrifuged at 10,000×g at room temperature for 10 min. The supernatant containing the solubilized protein was dialyzed in a cellulose membrane tube (20/32, Sanko Junyaku Co., Ltd., Tokyo, Japan) against a dialyzing buffer (20 mM Tris-HCl, pH 8.5) supplemented with 0.1 mM of DTT) at 4°C overnight through two changes of the buffer. Dialysis was continued in a dialyzing buffer without DTT for 6 h (3 h each) and then in redox refolding buffer (dialysis buffer supplemented with 0.2 mM oxidized glutathione and 1 mM reduced glutathione) at 4°C overnight to refold the protein and to promote disulfide bond formation. The sample was further dialyzed for a further 3 h at room temperature to enhance disulfide exchange rates. These processes were conducted according to the

instructions in the Protein Refolding Kit (Novagen).

*Ni column purification of a recombinant NM-Igl protein*

His-tagged refolded NM-Igl was purified using a Ni column. The protein solution (5 ml) was batched with 1 ml of gel slurry of Ni-NTA agarose (Qiagen) at 4°C overnight. The mixture was loaded onto hand-made columns. After collecting the flow through fraction, the column was washed three times with 1 ml of PBS containing 10 mM imidazole. Recombinant NM-Igl protein was eluted with PBS containing 200 mM imidazole (1 ml/fraction). Five eluted fractions from each run were collected and applied to SDS-PAGE gels. Fractions containing the recombinant protein were pooled and buffers were changed to PBST (PBS with 0.05% Tween 20) with Amicon Ultra Ultracel-10K (Millipore) before use in further studies. Protein concentrations were measured using BioRad protein assay reagent.

*SDS-PAGE and Coomassie Brilliant Blue staining of purified recombinant proteins*

Recombinant proteins (1 μg each) were mixed with a one-third volume of SDS sample buffer (Invitrogen) and subjected to SDS-PAGE using NuPAGE Novex Bis-Tris (4-12% gradient) gel in reducing condition. Electrophoresis was conducted for 40 min at 200 V. For Coomassie staining, the gel was treated with SimplyBlue Safe stain solution (Invitrogen) and incubated until blue bands appeared on the gel. The gel was washed with distilled water and photographs were taken.

*Hemolytic assay using recombinant lectins*

NM-Igl or C-Igl (2 μM) in PBST (total volume 50 μl) was loaded on to a 96U Nunclon microwell plate (Nunc). HoRBCs in PBS (2% v/v) were prepared as described above and 50 μl/well of the RBC solution was added to the Igl-preloaded wells at room temperature.

Hemolytic activities of the Igls were evaluated after RBC loading and images were taken at several time points.

*Detection of released hemoglobin concentration*

A Hemoglobin B Test Kit (Wako, Osaka, Japan) was used to quantify the hemolytic activity of each recombinant protein. The concentration of hemoglobin released by hemolysis in the supernatants of RBCs and in the recombinant protein mixtures described above were measured after incubation for 8 h. The results are expressed as a mean of 5 experiments with the standard deviation (SD).

*Trypan blue exclusion assay of F-Igl treated Caco-2 cells*

Caco-2 cells (ATCC, HTB-37) were cultured in MEM basic medium (Gibco) supplemented with Earle's salts, L-glutamine and 20% fetal bovine serum. After detachment with 0.25% Trypsin-EDTA (Gibco), the cells were cultured in a 96-well plate at approximately $2\times10^4$ cells/100 μl/well at 37°C under 5% $CO_2$ for 24 h. Then the medium was changed to 100 μl of medium with 1 μM F-Igl or PBST containing 200 units/ml penicillin G and 200 μg/ml streptomycin (Wako, Japan). The cells were incubated for an additional 1 or 12 h under the same conditions. After collecting the culture supernatants, 100 μl of fresh medium with 5 μl of 0.4% trypan blue was loaded to each well and the viability of attached cells was assessed. One microliter of 0.4% trypan blue was added to the collected culture supernatant and the viability of detached cells was also assessed. Those samples were observed under EVOS XL microscopy.

*Lectin activity assay using neoglycoprotein coated plate*

Tn Antigen-HSA, human serum albumin conjugated with the average of 19 GalNAc

residues per a molecule (Dextra Laboratories, UK), was coated to a F96 MAXISORP plate (Nunc, Denmark) at 10 pmol/well at 4°C overnight. HSA (Sigma Aldrich, St. Louis, MO) was used as a control. After washing with TBST for 3 times, the plate was blocked with 3% BSA at 4°C overnight. Two-fold serial diluted recombinant Igls in TBST (from 50 pmol/well) were loaded on to the plate after washing the plate with TBST and the plate was incubated at 4°C overnight. Bound recombinant Igls were detected after incubated with a mouse anti-His tag antibody (Novagen, San Diego, CA; 1: 1000 dilution in TBST), a HRP conjugated rabbit anti-mouse IgG (H+L) (abcam, Tokyo, Japan; 1: 5000 dilution in TBST) and 1-step ultra TMB ELISA solution (Thermo, Rockford, IL). The reaction was stopped by adding 50 μl of 1 M $H_2SO_4$ and absorbance of the samples at 450 nm was measured. Two-fold serial diluted recombinant Igls (from 10 pmol/well) were coated to a F96 MAXISORP plate and detected by the same antibodies to confirm that the same concentrations of the Igls were equivalently detected in this assay.

*Comparison of amino acid sequences of Igls with parasitic pore-forming proteins and an invertebrate hemolytic lectin*

Amino acid sequences of amoebapore-A (PDB: 1OF9_A)[30], acanthaporin (PDB: 2LRD_A)[31], and CEL-III (PDB: 1VCL)[35] were aligned and compared with those of C1-, C2-, and C3-Igl using CLUSTALW (http://clustalw.ddbj.nig.ac.jp/). Conserved amino acids between two sequences are shown in red bold characters. In the comparison of amoebapore-A with the Igls, conserved amino acids with NK-lysin are underlined.

**Figure Legends for Supplementary Data**

**Supplementary Figure S1. Lectin activities of F-Igl toward glycans on a glycan array**

(a) Signal-to-noise ratios (S/N) of affinities of F-Igl for glycans on the array. S/N values >3

were considered to be significant. These results showed no detectable affinity of F-Igl to the glycans. (b) The array was coated with 28 types of glycans.

**Supplementary Figure S2. Dose response experiments for hemagglutinating and hemolytic assays**

Two-fold serial diluted lectins (from 200 to 6.25 μg/ml) were mixed with HoRBCs (2% v/v). Hemagglutinating activity (1 h) and hemolytic activity (18 h) of the lectins were observed.

**Supplementary Figure S3. Major hemolytic activity in the C-terminus domain of Igl**

(a) NM-Igl was designed to contain both N-Igl and M-Igl. (b-d) Comparison of hemolytic activities. (b) NM-Igl or C-Igl (1 μg) was loaded onto a 4-12% Bis-Tris gel and checked for amount and purity. (c) A time-course hemolysis assay was conducted for NM-Igl and C-Igl. (d) Released hemoglobin concentrations were measured after 8 h of incubation. $^{**}p<0.01$ for PBST vs. Igl treatment (Dunnett's test).

**Supplementary Figure S4. Assessment of the viability of F-Igl treated Caco-2 cells**

(a) The viability of attached Caco-2 cells remained on the wells of the plates were assessed. Arrows indicate dead cells which could not exclude trypan blue. Bars indicate 100 μm.

(b) The viability of detached Caco-2 cells in culture supernatants was assessed. Live cells were observed in PBST treated cells while cluster of dead cells were observed in F-Igl treated cells. Bars indicate 50 μm.

**Supplementary Figure S5. Lectin activity assay using neoglycoprotein coated plate**

(a) Dose dependent lectin activities of F-Igl and C-Igl toward $(GalNAc)_{19}$-HSA were observed. An inset showed that the same concentrations of recombinant Igls were

equivalently detected in this assay. (b) All the recombinant Igls had a high affinity toward HSA dose dependently.

**Supplementary Figure S6. Amino acid sequence alignment of amoebapore-A with Igls**

Comparison of the amino acid sequence of amoebapore-A with those of C1-Igl (a), C2-Igl (b), and C3-Igl (c) using CLUSTALW. Amino acids in red are conserved between the proteins. Underlined amino acids are conserved between amoebapore-A and NK-lysin[24].

**Supplementary Figure S7. Amino acid sequence alignment of acanthaporin with Igls**

Comparison of the amino acid sequence of acanthaporin with those of C1-Igl (a), C2-Igl (b), and C3-Igl (c) using CLUSTALW. Amino acids in red are conserved between the proteins.

**Supplementary Figure S8. Amino acid sequence alignment of CEL-III with Igls**

Comparison of the amino acid sequences of CEL-III with those of C1-Igl (a), C2-Igl (b), and C3-Igl (c) using CLUSTALW. Amino acids in red are conserved between the proteins. (d) Location of the seven $CX_8C$ motifs in C-Igl in equivalent regions in Hgls and Igls. The motifs are not found in Hgls.

Novel hemagglutinating, hemolytic and cytotoxic activities of the intermediate subunit of *Entamoeba histolytica* lectin
Kentaro Kato, Kazuhide Yahata, Bhim Gopal Dhoubhadel, Yoshito Fujii, and Hiroshi Tachibana

Supplementary Figure S1

(a)

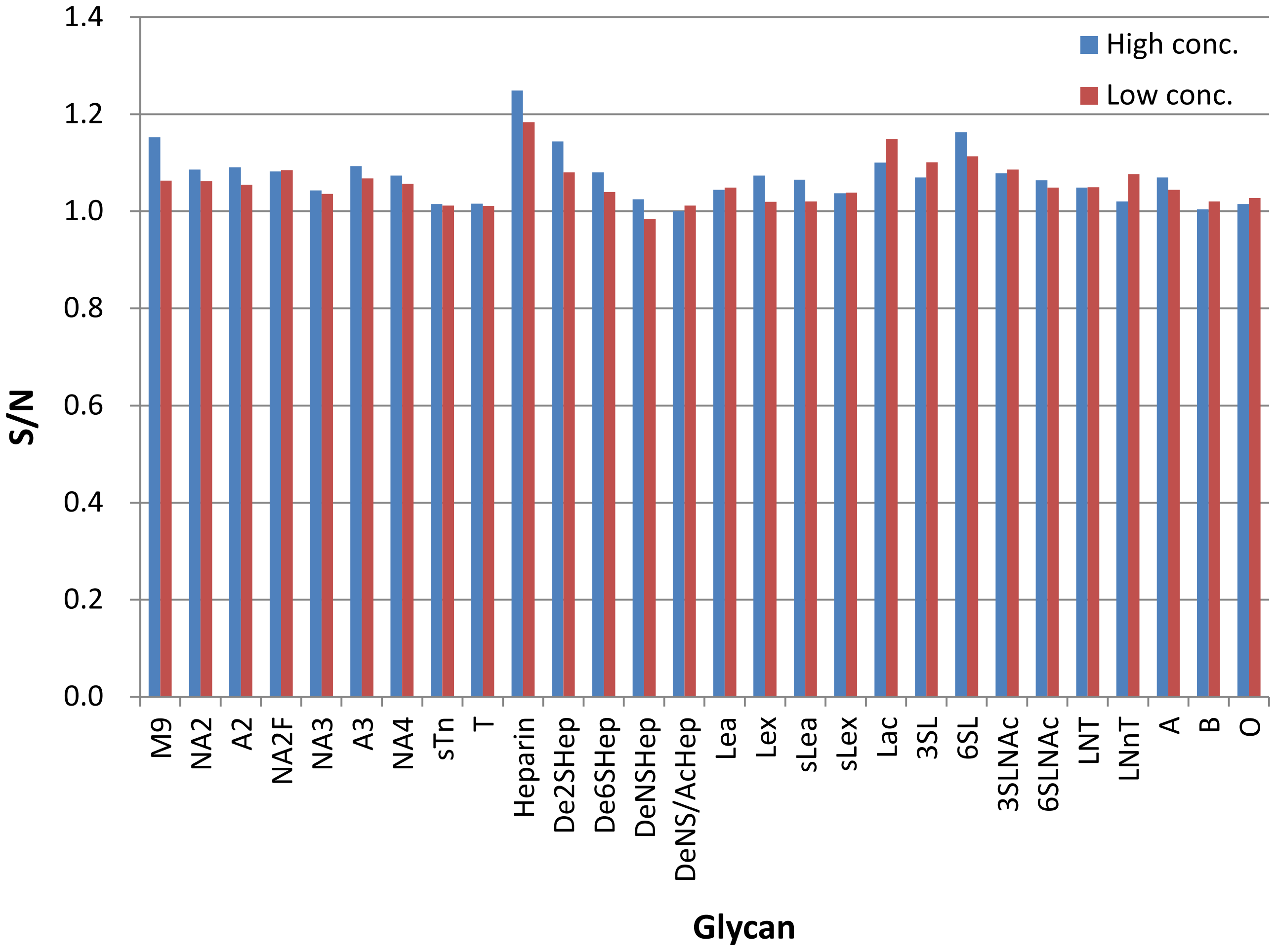

Kato K et al.
Supplementary Figure S1

(b)

[*N*-glycan]

1. M9

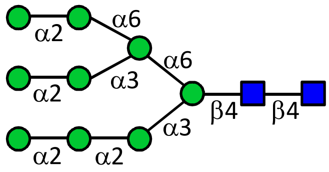


2. NA2

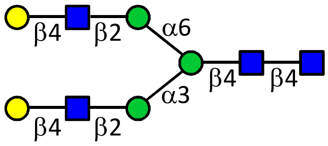


3. A2

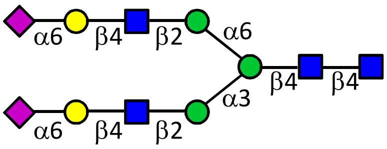


4. NA2F

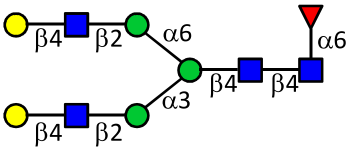


5. NA3

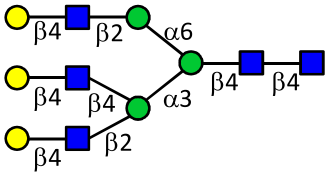


6. A3

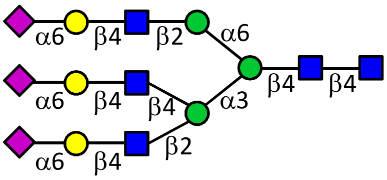


7. NA4

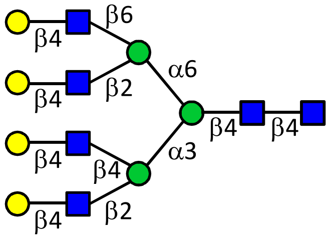


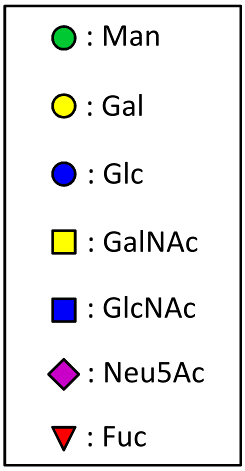


[*O*-glycan]

8. sTn

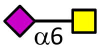


9. T

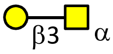


[Glycosaminoglycan (GAG)]

10. Heparin

11. 2-*O*-Desulfated Heparin

12. 6-*O*-Desulfated Heparin

13. *N*-Desulfated Heparin

14. *N*-Desulfated re*N*-Acetylated Heparin

Kato K et al.
Supplementary Figure S1

(b) continued

[Lewis type]

15. Lea

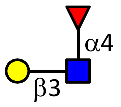


16. Lex

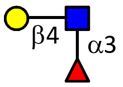


17. sLea

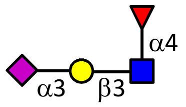


18. sLex

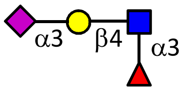


[Lac]

19. Lac

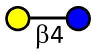


20. α2-3 Sialyl Lactose

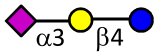


21. α2-6 Sialyl Lactose

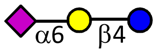


22. α2-3 Sialyl LacNAc

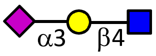


23. α2-6 Sialyl LacNAc

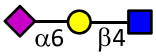


24. Lacto-*N*-tetraose

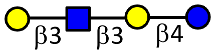


25. Lacto-*N*-neo-tetraose

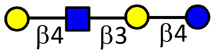


[ABO blood type]

26. A trisaccharide

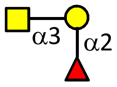


27. B trisaccharide

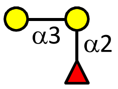


28. H(O) disaccharide

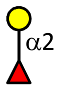

Kato K et al.
Supplementary Figure S2

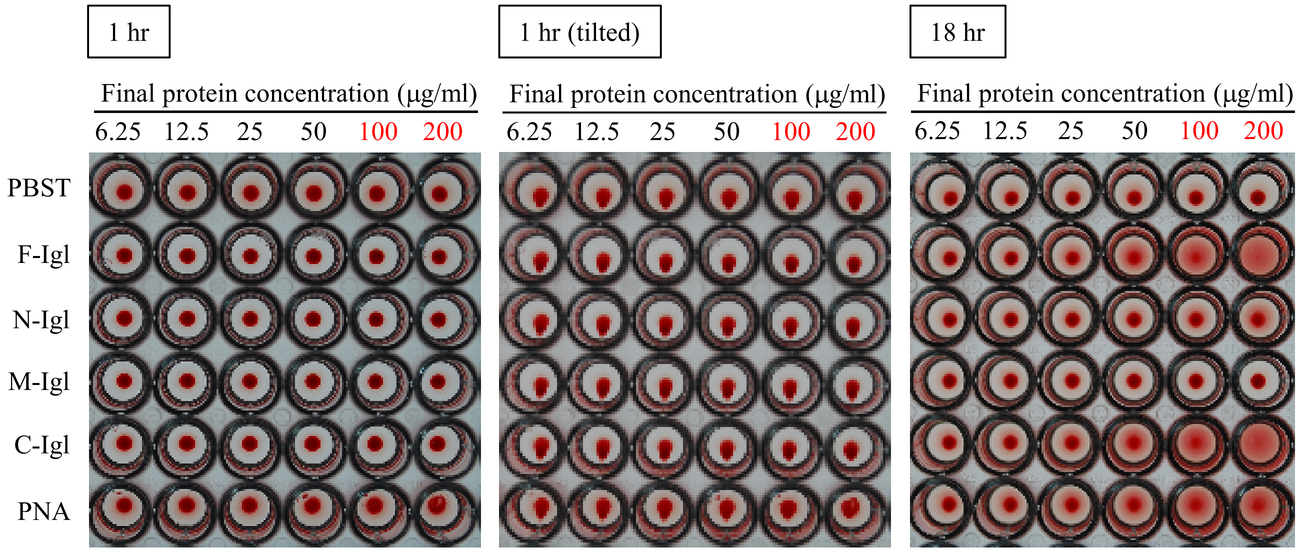

Kato K et al.
Supplementary Figure S3

(a)

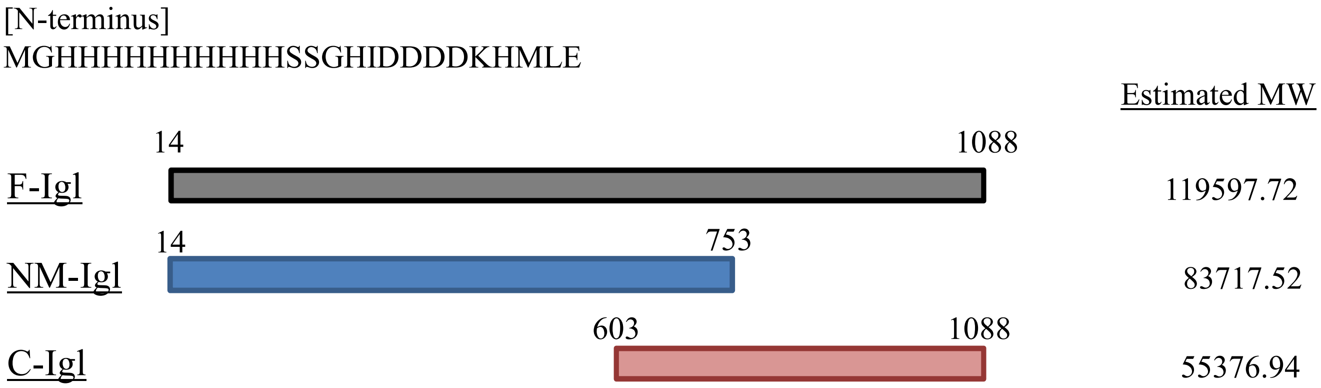


(b)

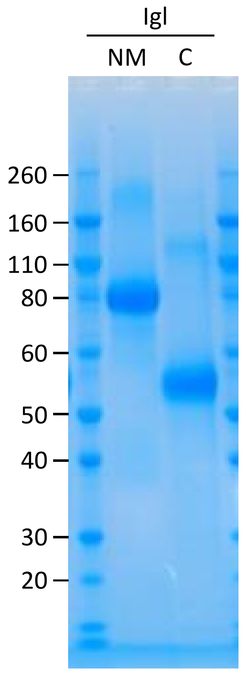

Kato K et al.
Supplementary Figure S3

(c)

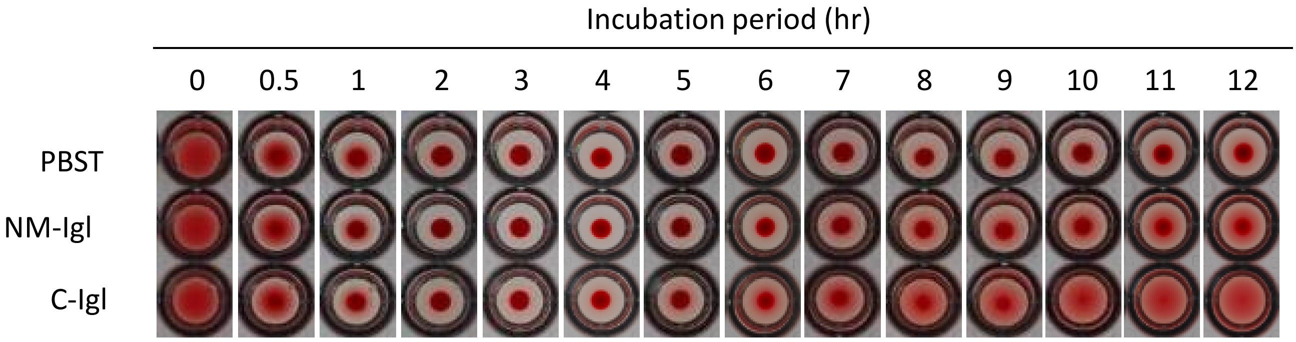


(d)

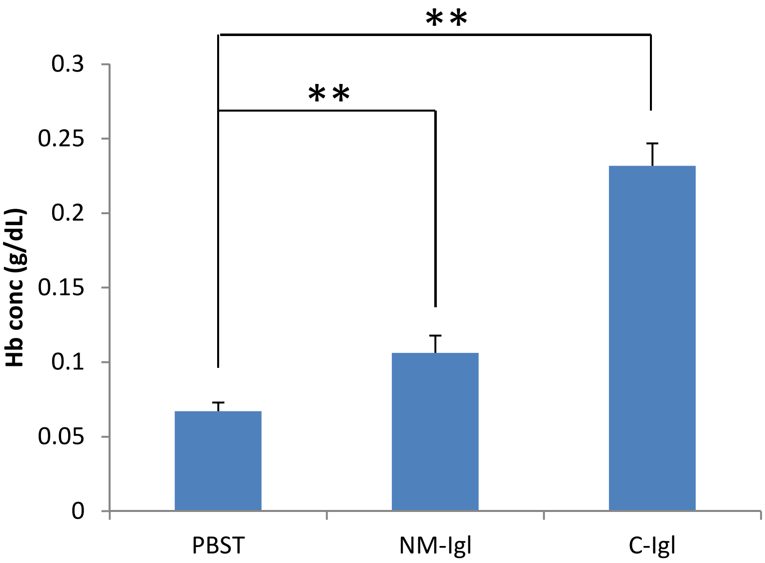

Kato K et al.
Supplementary Figure S4

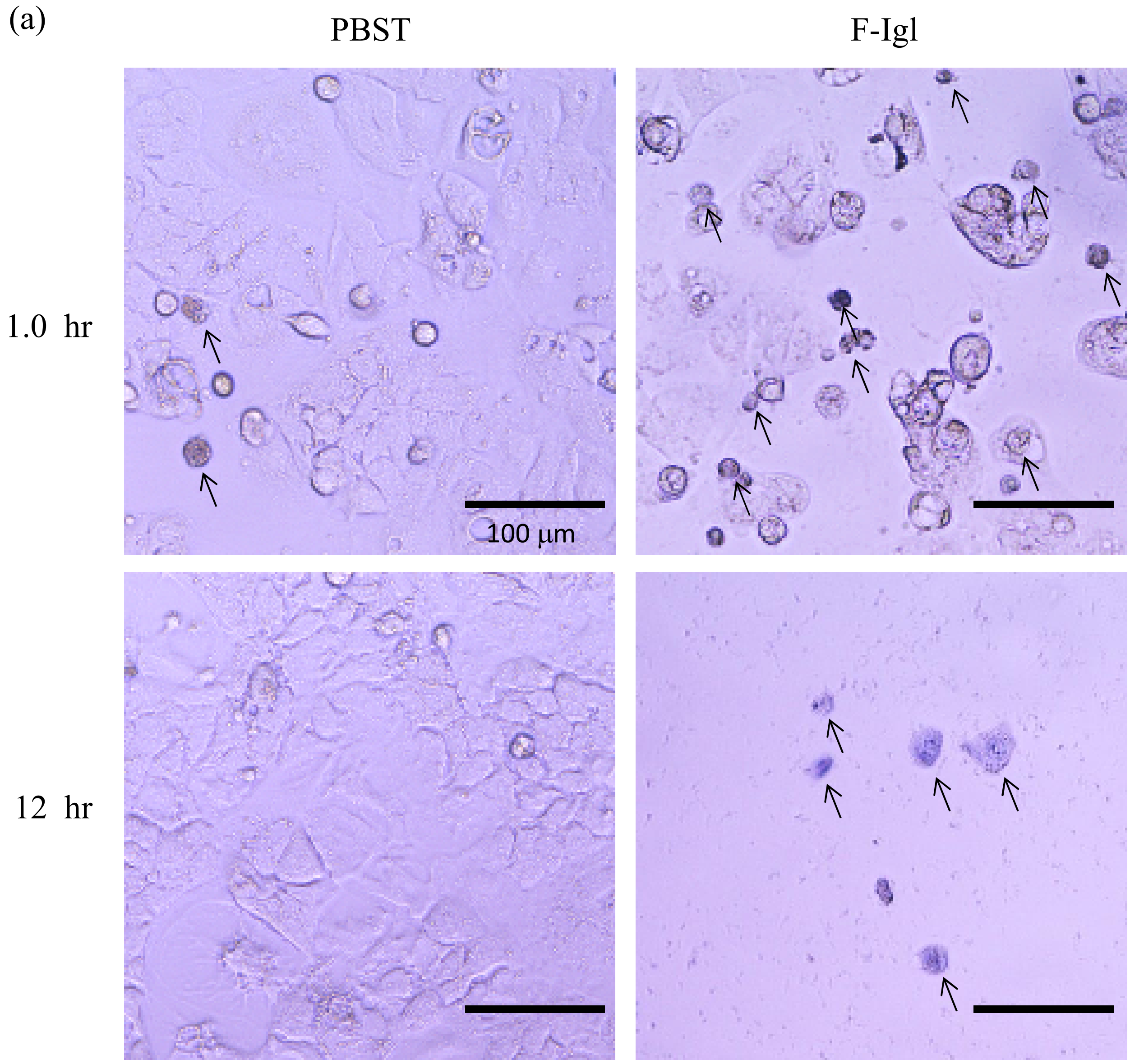

Kato K et al.
Supplementary Figure S4

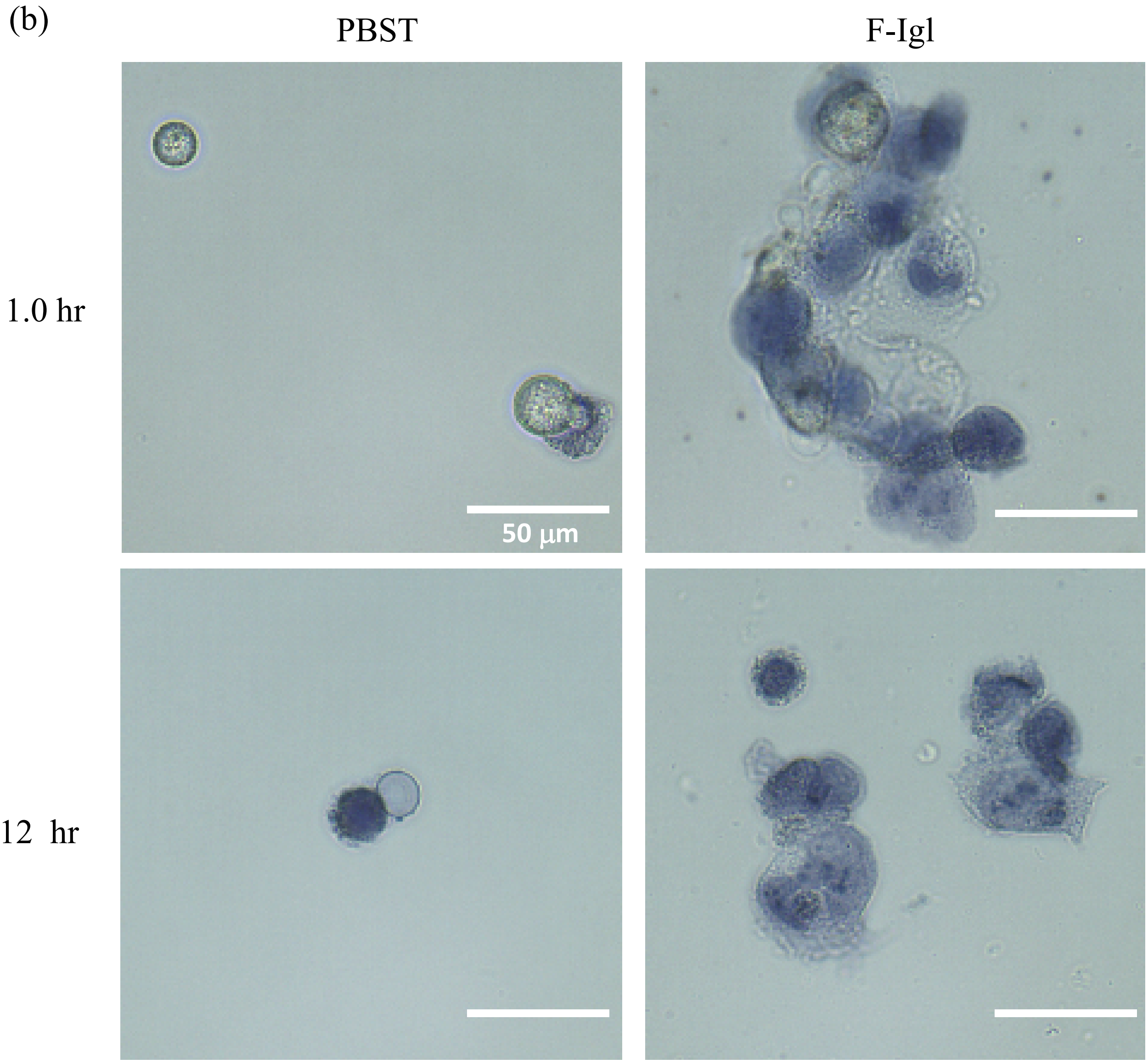

Kato K et al.
Supplementary Figure S5

(a)

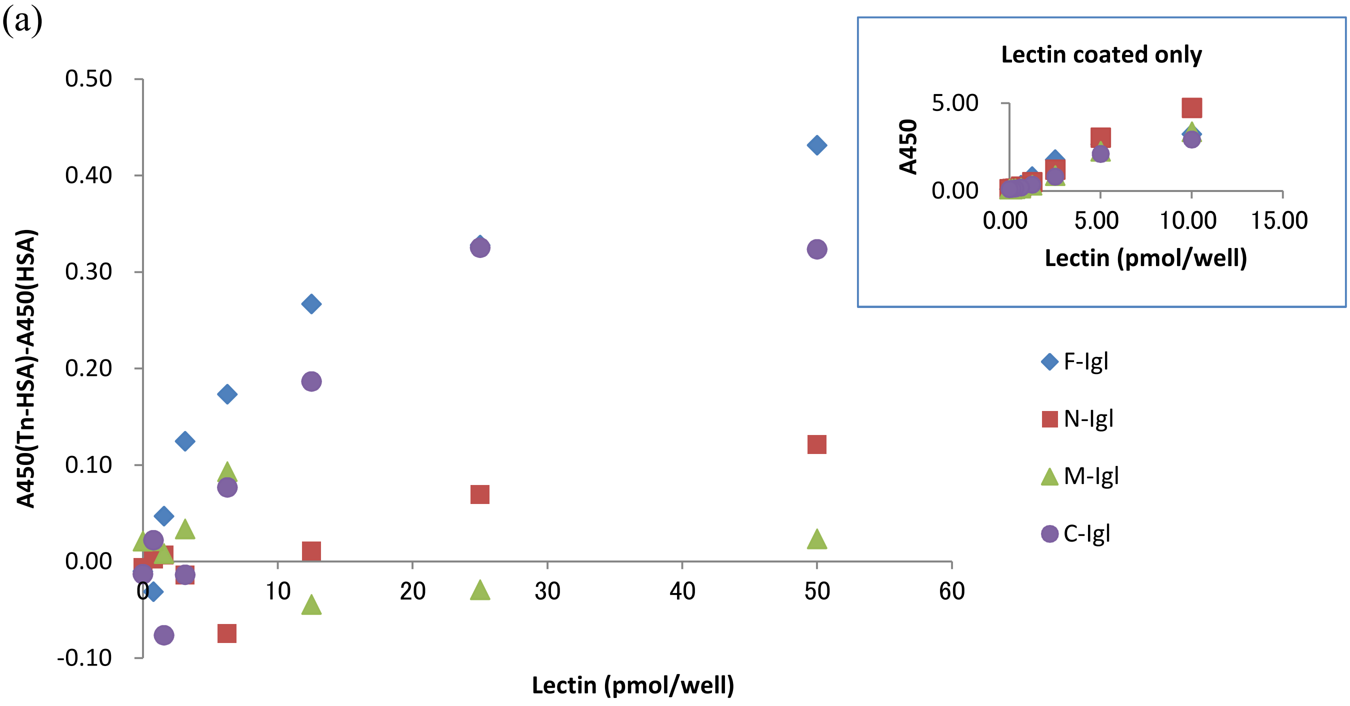


(b)

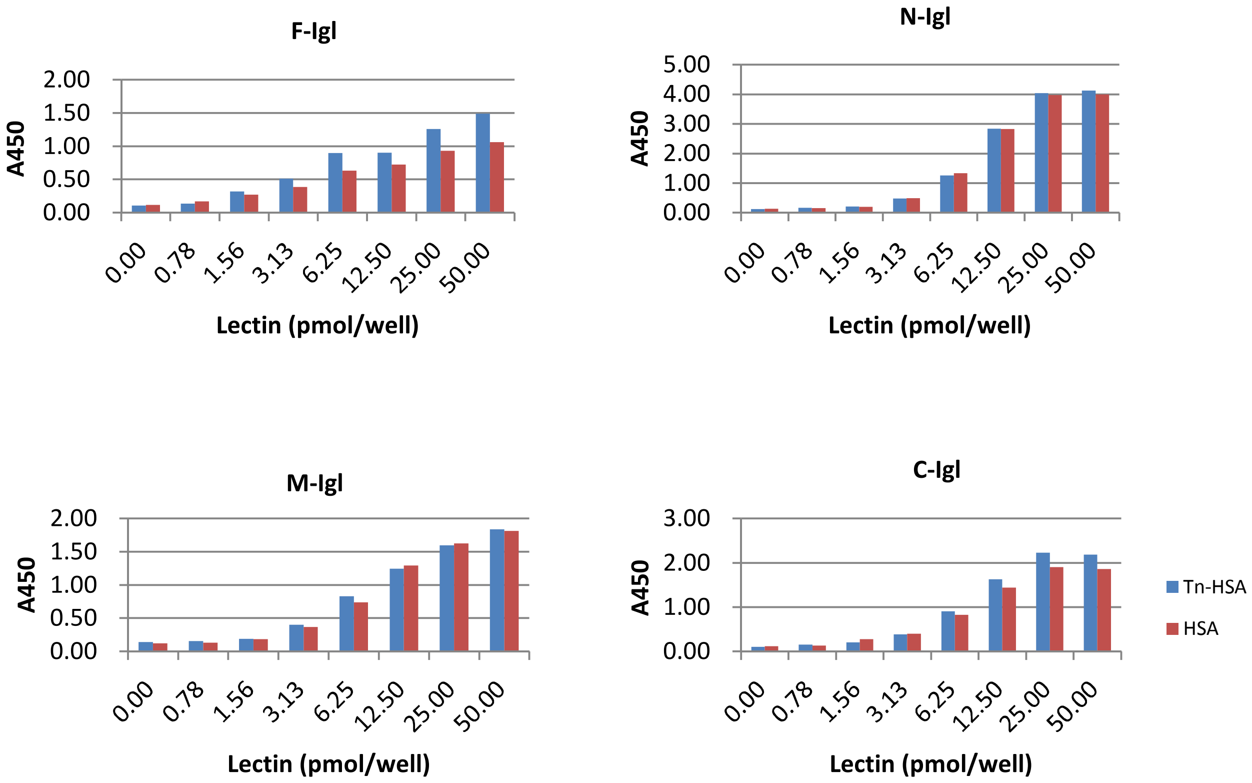

Kato K et al.
Supplementary Figure S6

(a)

```
                    10         20         30         40         50         60
C1-IgI      EGPNAEDKKK SCAQLNNNCK KEGKYEISDG FVTCLDCDDS AYIVGSQVGA CTQCSPNAFK
AmoebaporeA ---------- ---------- ---------- ---------- ---------- ----------

                    70         80         90        100        110        120
C1-IgI      DENNKCQLCS TKQSQYGHCA ACSATACITC EDINLILTGE KPCTVCKDGF YQIENATDGV
AmoebaporeA ---------- ---------- ---------- --------GE ILCNLCTGLI N---------

                   130        140        150        160        170        180
C1-IgI      YCSPCPAKCK TCKYNTTSKK VECVTCTEQR LKDIKAPECA CPTGTVQLEN GTCQSCSDLS
AmoebaporeA ---------T LENLLTTKGA D--------- ---------- ---------- ----------

                   190        200        210        220        230        240
C1-IgI      KYPGCKKTDS CNVDSRTGFI YATECSDGFS GRSPYSNCTT CTKSNYYPKE GEKNGCAKCD
AmoebaporeA ---KVKDYIS SLCNKASGFI -ATLCTKVLD FGI------- --DKLIQLIE DKVDANAICA

             244
C1-IgI      DKCA-
AmoebaporeA KIHAC
```

(b)

```
                    10         20         30         40         50         60
C2-IgI      PCPAKCKTCK YNTTSKKVEC VTCTEQRLKD IKAPECACPT GTVQLENGTC QSCSDLSKYP
AmoebaporeA ---------- -----GEILC NLCT------ ---------- ---------- ---GLINTLE

                    70         80         90        100        110        120
C2-IgI      GCKKTDSCNV DSRTGFIYAT ECSDGFSGRS PYSNCTTCTK SNYYPKEGEK NGCAKCDDKC
AmoebaporeA NLLTTKGADK VKDYI----- ---------- ---------- ---------S SLCNKASGFI

                   130        140        150        160        170        180
C2-IgI      ATCSDKDTCL TCADPLKVGS KCDGCKTGYY MSNGECKPCT NHCSECSSAA ECTVCESDTY
AmoebaporeA ATLCTKVLDF ------GIDK LIQLIEDKVD AN-------- ---------- ----------

                   190        200        210        220        230        240
C2-IgI      KVISGNGCNS CVDGFYFDEI KGTCIPCTSP CTKCVGVKKD CEEQETGCNS EKKKIVEECT
AmoebaporeA -AICAKIHAC ---------- ---------- ---------- ---------- ----------

           242
C2-IgI      KC
AmoebaporeA --
```

Kato K et al.
Supplementary Figure S6

(c)

```
                     10         20         30         40         50         60
C3-IgI      TCSDKDTCLT CADPLKVGSK CDGCKTGYYM SNGECKPCTN HCSECSSAAE CTVCESDTYK
AmoebaporeA ---------- --GEILCNLC TGLINTLENL LT-------- ----TKGADK VKDYISSLCN

                     70         80         90        100        110        120
C3-IgI      VISGNGCNSC VDGFYFDEIK GTCIPCTSPC TKCVGVKKDC EEQETGCNSE KKKIVEECTK
AmoebaporeA KASGFIATLC TKVLDFGI-- ------DKLI QLIEDKVDAN ---------- ----------

                    130        140        150        160        170        180
C3-IgI      CSTKDHIAEV PVNGACVCAY GYVEGTSTED NKIECQACKA KVNEFCDSCN SKDCLRCNAE
AmoebaporeA ---------A ICAKIHAC-- ---------- ---------- ---------- ----------

                    190        200        210        220        230        240
C3-IgI      YLEAKGGECV CVEGYYTSSW GSCIPCSRHM PHCTKCTGEG ECTTCEDGWK LKDGKCNGAK
AmoebaporeA ---------- ---------- ---------- ---------- ---------- ----------

           242
C3-IgI      GI
AmoebaporeA --
```

Kato K et al.
Supplementary Figure S7

(a)

```
                       10         20         30         40         50         60
C1-IgI         EGPNAEDKKK SCAQLNNNCK KEGKYEISDG FVTCLDCDDS AYIVGSQVGA CTQCSPNAFK
Acanthaporin   ---------- ---------- ---------- ---------- ---------GK CS-----VLK

                       70         80         90        100        110        120
C1-IgI         DENNKCQLCS TKQSQYGHCA ACSATACITC EDINLILTGE KPCTVCKDGF YQIENATDGV
Acanthaporin   --KVACAAAI A-----GAVA ACGG------ --IDL----- -PCVLA--AL ----KAAEG-

                      130        140        150        160        170        180
C1-IgI         YCSPCPAKCK TCKYNTTSKK VECVTCTEQR LKDIKAPECA CPTGTVQLEN GTCQSCSDLS
Acanthaporin   -CASC----- ---F------ -----CEDH- --------C- --------H- GVCK---DL-

                      190        200        210        220        230        240
C1-IgI         KYPGCKKTDSCNVDSRTGFIYATECSDGFSGRSPYSNCTTCTKSNYYPKEGEKNGCAKCD
Acanthaporin   --------HLC-------------------------------------------------

                244
C1-IgI         DKCA
Acanthaporin   ----
```

(b)

```
                       10         20         30         40         50         60
C2-IgI         PCPAKCKTCK YNTTSKKVEC VTCTEQRLKD IKAPECACPT GTVQLENGTC QSCSDLSKYP
Acanthaporin   ---GKCS--- ---VLKKVAC AAA----IA- ---------- -------GAV AACGGID-LP

                       70         80         90        100        110        120
C2-IgI         GCKKTDSCNV DSRTGFIYAT ECSDGFSGRS PYSNCTTCTK SNYYPKEGEK NGCAKCDDKC
Acanthaporin   -------C-- -----VLAAL KAAEG----- ----CASC-- ---F------ -----CEDHC

                      130        140        150        160        170        180
C2-IgI         ATCSDKDTCL TCADPLKVGS KCDGCKTGYY MSNGECKPCT NHCSECSSAA ECTVCESDTY
Acanthaporin   H--G-----V -CKD-LHL-- -C-------- ---------- ---------- ----------

                      190        200        210        220        230        240
C2-IgI         KVISGNGCNS CVDGFYFDEI KGTCIPCTSP CTKCVGVKKD CEEQETGCNS EKKKIVEECT
Acanthaporin   ---------- ---------- ---------- ---------- ---------- ----------

              242
C2-IgI         KC
Acanthaporin   --
```

Kato K et al.

Supplementary Figure S7

(c)

```
                      10         20         30         40         50         60
C3-IgI         TCSDKDTCLT CADPLKVGSK CDGCKTGYYM SNGECKPCTN HCSECSSAAE CTVCESDTYK
Acanthaporin   ---------- ---------- ---------- ---------- ---------- ----------

                      70         80         90        100        110        120
C3-IgI         VISGNGCNSC VDGFYFDEIK GTCIPCTSPC TKCVGVKKDC EEQETGCNSE KKKIVEECTK
Acanthaporin   ---------- ---------- ---------- ---------- ---------- ----------

                     130        140        150        160        170        180
C3-IgI         CSTKDHIAEV PVNGACVCAY GYVEGTSTED NKIECQACKA KVNEFCDSCN SKDCLRCNAE
Acanthaporin   ---------- ---GKCS--- ---------- -VLKKVACAA AIAGAVAACG GID-LPCVLA

                     190        200        210        220        230        240
C3-IgI         YLEAKGGECV CVEGYYTSSW GSCIPCSRHM PHCTKCTGEG ECTTCEDGWK LKDGKCNGAK
Acanthaporin   ALKAAEG--- CAS------- --CF-CEDH- --C-----HG ---VCKD-LH L----C----

             242
C3-IgI         GI
Acanthaporin   --
```

Kato K et al.
Supplementary Figure S8

(a)

```
                    10         20         30         40         50         60
C1-IgI      EGPNAEDKKK SCAQLNNNCK KEGKYEISDG FVTCLDCDDS AYIVGSQVGA CTQCSPNAFK
CEL-III     -------NGK VSQQISN--- -------TIS FSSTVTAG-- ---VAVEVSS TIEKG-VIFA

                    70         80         90        100        110        120
C1-IgI      DENNKCQLCS TKQSQYGHCA ACSATACITC EDINLILTGE KPCTVCKDGF YQIENATDGV
CEL-III     KASVSVKVTA SLSKAWTNSQ SGTTAITYTC D--N--Y--- ----D----- -----S-DEE

                   130        140        150        160        170        180
C1-IgI      YCSPCPAKCK TCKYNTTSKK VECVTCTEQR LKDIKAPECA CPTGTVQLEN GTCQSCSDLS
CEL-III     FT-------R GCMWQLAIET TEVK------ ---------- --SGDLLVWN PQIIKCTRSN

              ①     190        200        210        220        230        240
C1-IgI      KYPGCKKTDS CNVDSRTGFI YATECSDGFS GRSPYSNCTT CTKSNYYPKE GEKNGCAKCD
CEL-III     TAPGCAP--- --------FT ---KCAN--- -----EDCTF CTDI------ ----------

             244
C1-IgI      DKCA
CEL-III     ----
```

(b)

```
                    10         20         30         40         50      ① 60
C2-IgI      PCPAKCKTCK YNTTSKKVEC VTCTEQRLKD IKAPECACPT GTVQLENGTC QSCSDLSKYP
CEL-III     ---------- ---------- ---------- ---------N GKVSQQISNT ISFSSTVT-A

                    70         80         90        100        110        120
C2-IgI      GCKKTDSCNV DSRTGFIYAT ECSDGFSGRS PYSNCTTCTK SNYYPKEGEK NGCAKCDDKC
CEL-III     GVAVEVSSTI EK--GVIFAK AS---VSVKV TASLSKAWTN S----QSGTT AITYTCDN--

              ②&③ 130        140        150        160   ④ 170   ⑤    180
C2-IgI      ATCSDKDTCL TCADPLKVGS KCDGCKTGYY MSNGECKPCT NHCSECSSAA ECTVCESDTY
CEL-III     --Y-DSDEEF TRGCMWQLAI ETTEVKSGDL LVWN---PQI IKCTRSNTAP GCAP-----F

                   190        200        210        220        230        240
C2-IgI      KVISGNGCNS CVDGFYFDEI KGTCIPCTSP CTKCVGVKKD CEEQETGCNS EKKKIVEECT
CEL-III     T--------K CAN------- --------ED CTFCTDI--- ---------- ----------

           242
C2-IgI      KC
CEL-III     --
```

Kato K et al.

Supplementary Figure S8

(c)

```
                 ③ 10         20         30         40        ④ 50 ⑤      60
C3-IgI     TCSDKDTCLT CADPLKVGSK CDGCKTGYYM SNGECKPCTN HCSECSSAAE CTVCESDTYK
CEL-III    ---------- ---------- ---------- -NGKVSQQIS NTISFSSTVT AGVAVEVSST

                   70         80         90        100        110        120
C3-IgI     VISGNGCNSC VDGFYFDEIK GTCIPCTSPC TKCVGVKKDC EEQETGCNSE KKKIVEECTK
CEL-III    IEKG------ ---VIFAKAS ---------- ---VSVKVTA SLSKAWTNSQ ----------

                  130        140        150        160        170        180
C3-IgI     CSTKDHIAEV PVNGACVCAY GYVEGTSTED NKIECQACKA KVNEFCDSCN SKDCLRCNAE
CEL-III    -------S-- ---GTTAITY ---------- ---TCDNYDS DE-EFTRGC- ---MWQLAIE

                  190        200        210      ⑥ 220⑦       230        240
C3-IgI     YLEAKGGECV CVEGYYTSSW GSCIPCSRHM PHCTKCTGEG ECTTCEDGWK LKDGKCNGAK
CEL-III    TTEVKSGDLL VWN------- ---------- PQIIKCTRSN TAPGCAPFTK CANEDCTFCT

         242
C3-IgI     GI
CEL-III    DI
```

(d)

```
               ①            ②&③            ④&⑤            ⑥&⑦

HgI1       CSLIETREKV    STVCQNDNSCPII   C-KFVQLTDDPSL   IVVSIGLFKTYQL
HgI3       CSLIETREKV    STVCQNDNSCPII   C-KFVQLTDDPSL   IVVSIGLFKTYQL
IgI1       CSDLSKYPGC    CATCSDKDTCLTC   CSECSSAAECTVC   CTKCTGEGECTTC
IgI2       CSDLSKYPGC    CATCSDKDTCLTC   CSECSSAAECTVC   CTKCTGEGECTTC
CX8C motif CXXXXXXXXC    CXXXXXXXXC      CXXXXXXXXC      CXXXXXXXXC
                            CXXXXXXXXC      CXXXXXXXXC      CXXXXXXXXC
```
